# Intracardiac echocardiography Chinese expert consensus

**DOI:** 10.3389/fcvm.2022.1012731

**Published:** 2022-10-06

**Authors:** Zhong Jingquan, Long Deyong, Chu Huimin, Fu Hua, Han Xuebin, Jiang Chenyang, Li Yan, Li Xuebin, Tang Min, Wang Zulu, Xue Yumei, Zhang Jinlin, Zhang Wei, Zhang Xiaochun, Zhou Daxin, Zhang Yun, Ma Changsheng, Paul C. Zei, Luigi Di Biase

**Affiliations:** ^1^Key Laboratory of Cardiovascular Remodeling and Function Research, Chinese Ministry of Education, Chinese National Health Commission and Chinese Academy of Medical Sciences, State and Shandong Province Joint Key Laboratory of Translational Cardiovascular Medicine, Department of Cardiology, Qilu Hospital, Cheeloo College of Medicine, Shandong University, Jinan, China; ^2^Department of Cardiology, Qilu Hospital (Qingdao), Cheeloo College of Medicine, Shandong University, Qingdao, China; ^3^Beijing Anzhen Hospital, Capital Medical University, Beijing, China; ^4^Ningbo First Hospital, Zhejiang University, Ningbo, China; ^5^West China Hospital, Sichuan University, Chengdu, China; ^6^The Affiliated Cardiovascular Hospital, Shanxi Medical University, Taiyuan, China; ^7^Sir Run Run Shaw Hospital of Zhejiang University, Hangzhou, China; ^8^Tangdu Hospital, The Fourth Military Medical University, Xi’an, China; ^9^Peking University People’s Hospital, Beijing, China; ^10^Fuwai Hospital of Chinese Academy of Medical Sciences and Peking Union Medical College, Beijing, China; ^11^General Hospital of Northern Theater Command, Shenyang, China; ^12^Guangdong Provincial People’s Hospital, Guangzhou, China; ^13^Wuhan Asian Heart Hospital, Wuhan, China; ^14^Zhongshan Hospital, Fudan University, Shanghai, China; ^15^Brigham and Women’s Hospital, Boston, MA, United States; ^16^Albert Einstein College of Medicine, Montefiore Medical Center, Bronx, NY, United States

**Keywords:** intracardiac echocardiography, arrhythmia, transseptal puncture, congenital heart disease, structural heart disease, left atrial appendage closure, device implantation and lead extraction, cardiomyopathy and pulmonary arterial hypertension

## Abstract

In recent years, percutaneous catheter interventions have continuously evolved, becoming an essential strategy for interventional diagnosis and treatment of many structural heart diseases and arrhythmias. Along with the increasing complexity of cardiac interventions comes ever more complex demands for intraoperative imaging. Intracardiac echocardiography (ICE) is well-suited for these requirements with real-time imaging, real-time monitoring for intraoperative complications, and a well-tolerated procedure. As a result, ICE is increasingly used many types of cardiac interventions. Given the lack of relevant guidelines at home and abroad and to promote and standardize the clinical applications of ICE, the members of this panel extensively evaluated relevant research findings, and they developed this consensus document after discussions and correlation with front-line clinical work experience, aiming to provide guidance for clinicians and to further improve interventional cardiovascular diagnosis and treatment procedures.

## Process of forming this consensus

The consensus was written by the “Chinese ICE Expert Group,” which includes experts in echocardiography, cardiac electrophysiology, congenital heart disease, valvular heart disease and so on. The consensus was initiated by Professor Zhang Yun and Ma Changsheng, and Professor Zhong Jingquan and Long Deyong were mainly responsible for the completion of the manuscript. We held three relevant meetings (2022.1.15, 2022.4.2, and 2022.4.23) regarding the consensus. Details of meetings are provided in the Table 1.

**TABLE 1:** Details of meetings.

Minutes of ICE Chinese expert consensus conference	
Time:	2022.1.15 Startup meeting
Location:	Jinan
Person:	14
Experts:	Zhang Yun, Zhong Jingquan, Zhang Jinlin, Chu Huimin, Li Yan, Tang Min, Ma Changsheng, Long Deyong, Jiang Chenyang, Zhang Wei, Fu Hua, Xue Yumei, Zhou Daxin, Zhang Xiaochun
Meeting Minutes:	This launch was the first symposium of the ICE expert consensus, which invited leading experts in electrophysiology and structural heart disease to discuss the direction and structure of the expert consensus. Different sections are also assigned to each participating expert to claim different sections for content writing. There was particularly intense discussion based on the setting of different sections, the wording and selection principles of the questions, the grade of recommendation, and the addition of quantitative tables. In particular, the popularity and practicability of consensus will be increased in the presentation of expression, selection of the source of illustrations, different standardized operations, highlighting clinical pathways, technical points, technical operations, image recognition and other modes. To make ICE ultrasound application technology more standardized, standardized, and prospective. It also combines overseas application experience with local use methods to develop field norms and standards that are more in line with the use of Chinese clinicians.
Meeting Summary:	(1) Add English version based on Chinese version consensus (2) Establishment of a writing expert group and members of the group of reviewing experts (voting and making recommendations) (3) Experts from the writing group lead different sections.

**Minutes of ICE Chinese expert consensus conference**	

Time:	2022.4.2 Reading Conference
Location:	Meeting online
Person:	9
Experts:	Zhong Jingquan, Zhang Jinlin, Chu Huimin, Ma Changsheng, Jiang Chenyang, Zhang Wei, Fu Hua, Xue Yumei, Zhang Xiaochun
Meeting Minutes:	Based on the division of labor of the kick-off meeting, this meeting will read and optimize the overall content focusing on the previously given division of labor, and inspect the integrity and integrity of the content in stages. Due to the large number of ICE application scenarios, the content presented in order to increase the overall and comprehensive nature of the consensus needs to be subdivided, so 8 contributing experts were raised to 15 to supplement the plate content: Wang Zulu – Application of ICE in Cryoablation; Li Xuebin – Application of ICE in Lead Extraction; Han Xuebin – Application of ICE in Pulmonary Arterial Hypertension; Xue Yumei – Application of ICE in Reduction of Catheter Ablation-associated Complications; Zhang Wei – Application of ICE in Congenital Heart Disease; Fu Hua – ICE Guided Catheter Ablation for Atrial Fibrillation Chu Huimin – Application of ICE in Left Atrial Appendage Closure.
Meeting Summary:	(1) Content writing of newly added plates (2) Refinement of recommendation grades and text refinement (3) Recommendation of the list of review experts after the completion of the manuscript

**Minutes of ICE Chinese expert consensus conference**	

Time:	2022.4.23 Review Meeting
Location:	Meeting online
Person:	42
Experts:	Zhang Yun, Zhong Jingquan, Zhang Jinlin, Chu Huimin, Li Yan, Tang Min, Ma Changsheng, Long Deyong, Jiang Chenyang, Zhang Wei, Fu Hua, Xue Yumei, Zhang Xiaochun, Xie Ruiqin, Xu Jian, Han Xuebin, Li Shufeng, He Jiangui, Zheng Liangrong, Wang Zulu, Shu Maoqin, Yuan Zuyi, Xu Yawei, Liu Yan, Liu Liwen, Zhang Xiaochun, Chen Minglong, Fan Jie, Liu Qiming, Liu Xu, Xia Yunlong, Jiang Tingbo, Li Xuebin, Zhu Wenqing, Dong Jianzeng, Li Shuyan, Wang Jianan, Kong Xiangqing, Tang Baopeng, Liu Xingpeng, Sang Caihua, Chen Mao
Meeting Minutes:	All sections have completed the writing of the content based on the recommendations of the launch meeting and the manuscript reading meeting. Invited 15 writing experts and nearly 30 reviewers to participate in this review, hoping to collect most of the special suggestions and endorsements in the field of electrophysiology and be more authoritative. In particular, extensive suggestions were listened to one by one in the recommendation section of recommendation grade and content: (1) The grade of recommendation was adjusted in order to be rigorous and in line with current evidence support. (2) The pictures selected in the text were selected from the cases in the expert clinic, optimized and streamlined. (3) The full text was optimized and uniformly worded. (4) Two overseas experts were invited to write and review the manuscript.
Meeting Summary:	(1) Invite two overseas experts as invited reviewers. (2) Picture pixel improvement, evidence level readjustment as recommended. (3) Translation of English manuscripts. (4) The revised version of this opinion shall be sent to all writing and reading experts to uniformly collect the suggestions on the final version and complete the revision.

## Overview of intracardiac echocardiography

### Definition and background of intracardiac echocardiography

Intracardiac echocardiography (ICE) is an ultrasound imaging technique able to perform real-time high-quality imaging and/or hemodynamic measurement of the heart and its adjacent tissues with an ultrasound probe placed at the tip of a catheter delivered into the cardiac chambers via peripheral vessels (1–3). In view of its ability to directly visualize the cardiac structures and reveal the anatomical relationship between various parts of the heart, ICE has been increasingly used to guide cardiac interventions and monitor intraoperative complications. It is an important adjunct in various cardiac interventions.

A catheter with an ultrasound transducer was first delivered into the cardiac chambers of a dog via the jugular vein to obtain endocardial echo images of the left and right ventricles as early as 1960 (4). Since then, scientists and engineers further developed early ultrasound probes to image intracardiac structures (5, 6). With the invention of the phased array ultrasound transducer and other devices in succession (7, 8), ICE has been increasingly applied to guide clinical practice such as transseptal puncture (9–11). In recent years, percutaneous catheter interventions have continuously evolved, becoming an essential strategy for interventional diagnosis and treatment of many structural heart diseases and arrhythmias. Along with the increasing complexity of cardiac interventions comes ever more complex demands for intraoperative imaging. ICE is well-suited for these requirements with real-time imaging, real-time monitoring for intraoperative complications, and a well-tolerated procedure (3, 12, 13). As a result, ICE is increasingly used many types of cardiac interventions. Given the lack of relevant guidelines at home and abroad and to promote and standardize the clinical applications of ICE, the members of this panel extensively evaluated relevant research findings at home and abroad, and they developed this consensus document after discussions and correlation with front-line clinical work experience, aiming to provide guidance for clinicians and to further improve interventional cardiovascular diagnosis and treatment procedures.

### Types of intracardiac echocardiography catheters

Intracardiac echocardiography catheters currently in use can be classified into the following two types by their technologic principles: the mechanical rotary ultrasound catheter and the phased array ultrasound catheter (14). The former, with a mechanical 360° rotary ultrasound transducer placed at its tip, provides circular sectional images perpendicular to its long axis. With a single ultrasound frequency, the catheter is only suitable for short-range imaging within 6-8 cm around the probe, instead of Doppler imaging. It is poorly maneuverable as it needs to be placed in the right atrium under the guidance of a long sheath. As a result, the catheter is now mainly used for electrophysiological studies, if at all (12, 15). The latter, composed of a handle and a catheter, is the most commonly used type of ICE catheter in clinical practice. The handle has three rows of knobs to manipulate the catheter to flex and fix in four directions: anterior (A), posterior (P), left (L), and right (R) (1–3, 12, 16). With a 64-element phased array ultrasound transducer placed at the tip, the catheter provides a 90° fan-shaped visual field by longitudinal scanning. With a variable ultrasound frequency (5–10 MHz) and a maximum penetration depth of 15–16 cm, the catheter is able to scan the cardiac chambers in all directions, along with Doppler imaging, via the manipulation of the handle. One currently utilized phased array ICE catheter integrates the 2D intracardiac US image with the 3D electroanatomical mapping system by embedding a position sensor at its tip. In this way, the ultrasound sector can be represented on the three-dimensional map to correlate the anatomical structures imaged with real-time catheter position (13), further improving the clinician’s understanding of intraprocedural cardiac anatomy in the context of planned interventions.

### Clinical applications of intracardiac echocardiography

Initially, ICE was mainly used to guide the interventional closure of atrial septal defect (ASD) and patent foramen ovale (PFO), with comparable image quality to that of transesophageal echocardiography (TEE). Nowadays, ICE is more common imaging modality used during these procedures, as it requires neither general anesthesia nor sonographer assistance (2, 13). In addition, ICE can image the interatrial septum, identify the location and anatomy of the fossa ovalis and assist proceduralists in selecting an ideal transseptal puncture site, and improve the overall success rate and safety of transseptal puncture (12). ICE is now used in a variety of interventional procedures requiring transseptal puncture, such as radiofrequency catheter ablation for atrial fibrillation (AF) or left ventricular arrhythmias, mitral valve intervention, and left atrial appendage closure (LAAC) (12). Surgeons are sometimes unable to precisely determine the relationship between the ablation target and a specific anatomical site because of the low resolution of 3D navigation and the 2D image overlay of fluoroscopy/radiography. However, ICE, as mentioned above, can clearly and accurately visualize the detailed and precise anatomical relationship between each cardiac chamber of interest in real time throughout the procedure, and assess the contact between the catheter and tissue, thereby improving the accuracy and efficacy of ablation. It plays an important role in the interventions of complicated arrhythmias such as atrial fibrillation, atrial flutter and ventricular arrhythmia (3, 12). In addition, with its ability to visualize the structures of the esophagus, arteries, atrial appendages, etc., ICE is expected to avoid or reduce the occurrence of complications, increase operators’ confidence, shorten the learning curve and increase success rates (17). Moreover, ICE is used to guide a variety of interventions and monitor related complications, such as transcatheter aortic valve replacement (TAVR), closure of patent ductus arteriosus (PDA), closure of para-valvular leak (PVL), closure of ventricular septal defect (VSD), balloon pulmonary valvuloplasty, radiofrequency ablation of the interventricular septum for hypertrophic obstructive cardiomyopathy, left ventricular pacing, interatrial septal pacing, interventricular septal pacing, pericardiocentesis, myocardial tissue biopsy, screening of intracardiac thrombus, and implantation and removal of cardiac implantable electronic devices (2, 3, 12, 15).

Early use of ICE was associated with a complication incidence of approximately 4%, mainly atrial tachycardia induced by the catheter manipulation in the right atrium (18). Despite its significant clinical application value, one factor restricting the wide clinical application of ICE is its high cost. However, data from a study in the United States indicated that the overall cost of intraoperative ICE is comparable to that of TEE (19). The overall cost-effectiveness and/or value added for ICE remains to be evaluated.

In conclusion, with the ability to image intracardiac structures and the adjacent anatomical relationship in real-time, ICE has gradually increased in use to guide interventional procedures for multiple structural heart diseases and arrhythmias and monitor intraoperative complications. Recommendations for the clinical application of ICE are shown in Table 2. Compared with X-ray and TEE, ICE has advantages of no radiation exposure, better tolerance, and no need for general anesthesia and sonographer assistance. Nowadays, 4D (real-time three-dimensional) ICE which can obtain high-quality 2D and 3D images in real time, has been gradually applied. In the future, ICE with higher image resolution and definition, reduced catheter diameter, and lower price, will very likely be even more widely used in a variety of clinical settings.

**TABLE 2:** Summary of recommended ICE applications.

Surgery/Procedure	Recommendation description	Recommended category
Transseptal puncture	The application of ICE guidance is recommended in the process of transseptal puncture. Particularly in patients with oversized or undersized atria or abnormal atrial septal anatomy or structures, thoracocyllosis, pectus excavatum, congenital cardiac anomolies etc. ICE-guided transseptal puncture is recommended, with expected increased procedural (TSP) success rates and decreased complications.	Recommended
Screening for atrial thrombus	ICE may replace TEE for screening patients for LAA thrombus who are reluctant to receive or not able to tolerate TEE, and whose atrial thrombus cannot be diagnosed or ruled out by CTA	Recommended
Catheter ablation of atrial fibrillation	ICE can be utilized to assist in guiding catheter ablation of atrial fibrillation to reduce the radiation exposure of patients and operators.	Recommended
Cryoballoon ablation for atrial fibrillation	ICE may be applied to guide balloon positioning and assesses PV occlusion occlusion, etc., to reduce the x-ray exposure time and the use of contrast medium when the non-balloon obstructive ablation is used.	Can be useful
Other atrial tachyarrhythmia	ICE may be used for imaging guidance during radiofrequency catheter ablation for premature atrial contractions, atrial tachycardia, atrial flutter, inappropriate sinus tachycardia, etc.	Can be useful
Catheter ablation for fascicular ventricular tachycardia	Assist in the identification of the origin of the ventricular tachycardia and judgment of catheter contact.	May be useful
Catheter ablation for ventricular arrhythmia originating from the outflow tract	ICE may be attempted to assist catheter ablation.	May be useful
Catheter ablation for ventricular arrhythmia originating from papillary muscles and regulatory tracts	ICE-assisted catheter ablation is recommended.	Recommended
Catheter ablation for ventricular arrhythmia originating from the left ventricular roof and other special heart chambers	ICE-assisted catheter ablation may be useful in guiding catheter-tissue contact, catheter manipulation.	May be useful
Catheter ablation for organic ventricular tachycardia	ICE may be used to assist in the identification of the myocardial matrix and scarred region with organic ventricular tachycardia and in the judgment of catheter contact and monitor ablation-related complications.	Can be useful
Monitoring of the complications during ablation	Help to monitor such complications as pericardial tamponade, acute thrombosis, as well as ablation injuries, to improve the overall surgical safety	Recommended
Occlusion of atrial septal defect and patent foramen ovale	ICE may be applied to guide the occlusion of secundum atrial septal defect and patent foramen ovale	Can be useful
Interventional procedure for ventricular septal defect	ICE may be used to guide interventional VSD closure in patients with complex anatomy.	Can be useful
Patent ductus arteriosus, etc.	ICE is recommended for interventional occlusion of PDA in patients with a large PDA, with renal insufficiency and allergies to contrast agents	Can be useful
Transcatheter aortic valve intervention	ICE may replace TEE in elderly aortic valve stenosis patients with esophageal lesions or not suitable for general anesthesia.	Can be useful
Transcatheter mitral valve intervention	ICE can be used to guide transseptal puncture and assess mitral regurgitation	May be useful
Transcatheter pulmonary valve intervention	ICE can be used in transcatheter pulmonary valve intervention to image the outflow tract of the right ventricle and pulmonary valve	Can be useful
Tricuspid valve invention	The ICE-guided procedure is recommended in patients with throat or esophageal lesions and those in whom anesthesia is contraindicated	Recommended
	3D/4D ICE-guided tricuspid valve invention	Can be useful
PVL intervention	ICE is applied to localize Perivalvular leak (PVL), help to select devices and determine the residual leak during the procedure and identify perioperative complications	Can be useful
	ICE-guided 3D/4D interventional procedure for PVL	Can be useful
Left atrial appendage occlusion	ICE may be used to evaluate structure and morphology of the left atrial appendage for guiding occlusion, selection of the best device type and size, assessing residual shunt, and related complications, etc.	Recommended
Radiofrequency catheter ablation in the ventricular septum for hypertrophic obstructive cardiomyopathy	ICE may be used to provide detailed anatomical information of the interventricular septum.	Recommended
Myocardial biopsy	ICE may be used for guiding the endomyocardial biopsy to reduce the risks associated with biopsy.	Can be useful
Left ventricular assist device implantation	ICE may be considered to guide the assist device implantation in the left ventricle when other imaging modalities are not appropriate	May be useful
Use in pregnant patients	ICE may be recommended preferentially to guide the conventional catheter ablation for tachyarrhythmia during pregnancy, pacemaker implantation, and interventional zero-ray therapy for some structural heart diseases.	Recommended
Preoperative examination or postoperative follow-up	ICE is performed in preoperative examination or postoperative follow-up	Not recommended
Removal of pacemaker electrode in high-risk cases	ICE is used for guiding lead extraction, reducing radiation exposure, and monitoring for operational complications	Can be useful

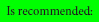
 With definite clinical benefits, ICE can be preferentially applied.

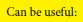
 Can be useful: With most clinical benefits and better efficacy, ICE can be applied in most cases.

May be useful: With insufficient evidence of clinical benefit, ICE may be applied based on the clinical realities.

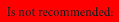
 Without clinical benefit or with clinical damage, ICE is not recommended.

## Application of intracardiac echocardiography in interventional diagnosis and treatment of arrhythmias

Intracardiac echocardiography helps the operator understand the key anatomy associated with arrhythmias, determine the spatial relationship between mapping and ablation catheters and their corresponding cardiac structures, and directly observe and guide the adjustment of degree of contact between the tip of the ablation catheter and the tissue. ICE can be used to monitor the formation, site, extent, and degree of ablation lesions to help determine the efficacy of ablation. Moreover, ICE can be used to monitor for complications in real-time to help determine their sites and severity. With the real-time monitoring function of ICE, complications can often be detected and managed before a hemodynamic change occurs. ICE can visualize the entire cardiac structure and accurately locate the aortic root and pulmonary sinus even when manipulated in the right heart. ICE is moreover instructive during ablation of arrhythmias originating from the outflow tracts, and it is critical for mapping and ablation of arrhythmias originating from protruding intracardiac structures, such as papillary muscle, false tendon, and moderator band. Furthermore, ICE allows observation of myocardial contraction, ventricular arrhythmia substrates such as scar/fibrosis, all the while enabling reduction of exposure to X-ray radiation and contrast agents.

### Application of intracardiac echocardiography in atrial arrhythmia

During interventional procedures for atrial arrhythmias, ICE can aid in the assessment of anatomical characteristics of the pulmonary veins (number, diameter, anatomical variation), the guidance of transseptal puncture, the screening for atrial/atrial appendage thrombus, and the monitoring of ablation lesion formation (3, 20). ICE can also monitor for and avoid possible complications in real-time, including esophageal thermal injury, inadvertent aortic puncture during TSP, and early detection of cardiac tamponade/thrombosis, so as to improve surgical safety (3). In addition, ICE is performed via an endovascular (venous) approach under local anesthesia, avoiding the risks of general anesthesia and discomfort from esophageal instrumentation (21). ICE is performed independently by the operator, thereby reducing labor costs (22). ICE-guided low X-ray or zero X-ray catheter ablation and LAAC have become increasingly mature and prevalent (23–25).

#### Application of intracardiac echocardiography in screening of left atrial appendage thrombus

Intracardiac echocardiography and TEE have distinct advantages and limitations. TEE has traditionally been the gold standard for the exclusion of left atrial and left atrial appendage thrombi and for anatomic delineation during catheter ablation for atrial fibrillation and LAAC. It is however, associated with increased patient discomfort and risk. Patients need to fast prior to the examination, and they need to be highly cooperative during the examination, and there is risk of esophageal injury during the examination. Left atrial CTA may also be used for the exclusion of left atrial appendage thrombi, but limitations include relatively high false-positive rates and the need for institutional experience for high quality images and accurate interpretation. IV contrast agent injection is required, with associated risks of anaphylaxis and kidney injury. Compared with TEE, ICE is expensive, as it uses a disposable catheter, but it is associated with less discomfort, greater compliance, lower incidence of complications, and less overall procedural radiation exposure. Many clinical studies evaluation ICE for left atrial and left atrial appendage thrombi have shown that (26–29). ICE is equivalent to TEE in clinical application. During ICE examinations, the left atrium and left atrial appendage can be scanned with the ICE catheter placed in the right atrium, coronary sinus ostium, right ventricular outflow tract, and pulmonary artery via a femoral venous approach (26). When the ICE probe is placed in the right ventricular outflow tract and pulmonary artery, the quality of left atrial appendage (LAA) imaging is significantly better, allowing effective identification of left atrial appendage thrombus. In contrast, when the ICE probe is placed in the right atrium, the quality of LAA imaging can be relatively poor (29). Since the coronary sinus ostium is close to the left atrial appendage, ICE placed in the coronary sinus can clearly show a cross-sectional view of the parallel left atrial appendage. The disadvantages of ICE include the following: The operation of the catheter tip is restricted in the coronary sinus, and the ICE catheter tip is relatively stiff and may associated with the risk of dissection or venous perforation if not performed properly (3).

Intracardiac echocardiography is a good option for the screening of left atrial and left atrial appendage thrombi in patients unable or unwilling to undergo TEE due to esophageal pathology, comorbidities rendering repeated sedation events riskier, or if left atrial CTA cannot confirm or rule out left atrial appendage thrombus. Further, ICE in patients who have undergone TEE or CTA, especially for when TEE suggests of “significant clouding,” “suspected thrombus,” and other unclear findings ICE may add additional diagnostic value (30, 31).

#### Application of intracardiac echocardiography in transseptal puncture

Transseptal puncture was first used for left atrial manometry by Ross et al. (32) in 1959 and rapidly popularized in the 1980s with the development of percutaneous balloon mitral valvuloplasty (PBMV) (33). Today, transseptal puncture has become essential in the process of cardiac interventional procedures such as catheter ablation of the left heart, intervention for congenital heart disease, LAAC and Left Ventricular Assist Device (LVAD) implantation. Transseptal puncture is traditionally performed under the primary guidance of conventional 2D fluoroscopy. Despite a high success rate (34), this approach has significant limitations. First, the specific procedure may dictate the optimal puncture site to facilitate catheter manipulation within the area of interest. Second, for patients with normal cardiac anatomy, a conventional fluoroscopy-guided transseptal puncture is safe and effective, but for patients with anatomical variations, which may not be known prior to the procedure the risk of puncture failure and complications may be significant. These risks include cardiac tamponade, puncture of the aortic root, arterial embolism, and pulmonary vein perforation.

Using ICE as the primary tool to guide transseptal puncture can make the puncture process easier, safer, and more specifically directed within the interatrial septum. Unlike TEE, ICE can be used in combination with the mapping system for 3D reconstruction by a single operator, with a wider field of view and no need for general anesthesia. One particular ICE system (Cartosound, Biosense Webster) enables contouring the cardiac structures visualized on ICE onto the EA map, as a magnet-enabled ICE catheter tip allows orientation of the catheter and hence ICE images within the 3D map. Before the transseptal puncture is performed, the operator can reconstruct key structures including the left atrium, fossa ovalis and aorta using ICE, and then select and mark the appropriate puncture site by adjusting the image sector as needed for subsequent steps. For the actual puncture procedure, ICE can visualize the entire TSP process. As a key step, when the puncture needle sheath is delivered into the fossa ovalis, a “tenting sign” will be observed at the fossa ovalis by ICE. Microbubbles seen using saline injection after the needle is inserted can help to further confirm the needle tip location in relation to the fossa ovalis. Proper entry of the needle tip into the left atrium can be confirmed by microbubble shadowing in the left atrium during saline injection through the needle (Flow chart 1 shown in Supplementary materials). Excessive needle insertion should be avoided (3, 25, 35, 36).

**FIGURE 1:**
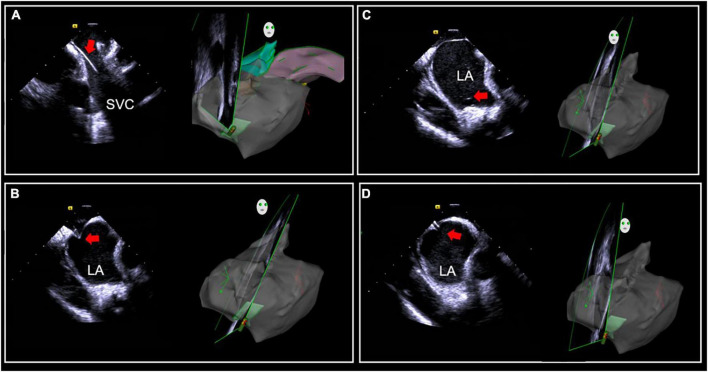
Intracardiac (ICE)-guided zero X-ray transseptal puncture. **(A)** Guidewire and sheath seen in SVC. **(B)** “Tenting sign” in the fossa ovalis by puncture needle sheath. **(C)** Saline injection microbubbles into the left atrium after the needle is inserted. **(D)** Needle tip into the left atrium. SVC, superior vena cava; LA, left atrium.

Intracardiac echocardiography is even more valuable for transseptal puncture in patients with abnormal anatomical structures, such as interatrial septal thickening and interatrial septal aneurysm, and it is quite valuable for transseptal puncture after cardiac surgery, and after interatrial septal closure. ICE can accurately determine the positional relationship between the needle sheath and fossa ovalis, select an appropriate puncture site, improve the success rates of puncture, and avoid serious complications (37, 38). Therefore, ICE guidance can be routinely considered in these patients. In addition, an entirely zero X-ray transseptal puncture can be achieved under real-time ICE guidance, which is of great significance for pregnant and pediatric patients with arrhythmias (Figure 1). However, the zero X-ray approach is only suitable for experienced operators; normally, the transseptal puncture should be done in conjunction with conventional radiography and ICE.

#### Intracardiac echocardiography-guided catheter ablation for atrial fibrillation

Intracardiac echocardiography plays an important role during the entire course of catheter ablation for atrial fibrillation, improving the comfort, safety and efficiency of the procedure. Even for pulmonary vein antrum isolation, ICE real-time image monitoring also helps to improve the safety and accuracy of the procedure.

As for point-by-point ablation solely based on the contours of the left atrium and pulmonary veins in fast anatomy mapping (FAM), there is often a spatial deviation between the planned ablation point and the real point. This may be caused by shifts in patient respiratory movement during the procedure, mapping inaccuracies, or anatomic-spatial changes resulting from changes in rhythm. However, with ICE two-dimensional imaging in combination with the Carto-Sound module to construct three-dimensional models of the left atrium and pulmonary veins in a 3D electroanatomical mapping system, the accuracy of FAM can be improved. In most clinical studies, the ultrasound catheter was placed in the right atrium. To further improve the model accuracy, the ICE catheter was then placed in the left atrium in some studies. In this way, the modeling was not only feasible but also more accurate (compared with modeling in the right atrium) (39). On this basis, some study sites performed zero X-ray radiofrequency ablation for atrial fibrillation with the integration of ICE and 3D electroanatomical mapping. As a result, 19 of 21 patients with atrial fibrillation received zero X-ray radiofrequency ablation throughout the entire course of the procedure (25). In addition, the procedure was safe and effective (40), without any procedure-related complications. In cryoablation for atrial fibrillation, PV occlusion assessment by ICE-guided balloon positioning can reduce the duration of X-ray exposure and the dose of a contrast agent, thereby improving the efficiency of the procedure.

Real-time three-dimensional ICE, or four-dimensional ICE, is a new technique emerging in recent years. Few studies have been reported on ICE-guided catheter ablation for atrial fibrillation. However, with the conduct of relevant research, we expect it to be useful in the whole process of catheter ablation for atrial fibrillation with improved efficiency and safety (Flow chart 2 shown in Supplementary materials).

#### Application of intracardiac echocardiography in other atrial arrhythmias

Intracardiac echocardiography is also useful during the ablation of other atrial arrhythmias similar to that in atrial fibrillation ablation, primarily including the screening of LA thrombus, delineation of anatomical structures, direct observation of the contact and movement of catheter relative to the endocardium and the change of focus on ablation during radiofrequency ablation, monitoring for thrombus during ablation, and possible prevention of steam pops. In addition, the examination process of ICE-guided ablation for left atrial-related atrial arrhythmias is similar to that of ICE-guided ablation for atrial fibrillation. For right atrial-related atrial arrhythmias, the ICE catheter can be placed in the right atrium to visualize important anatomical structures such as the tricuspid isthmus, tricuspid annulus, right atrial appendage, coronary sinus ostium, and superior vena cava by rotating and flexing the catheter clockwise or counterclockwise from the “home-view.” Several articles have suggested that ICE can visualize such anatomical structures as trabecula, depression, and Eustachian valve above the tricuspid isthmus line for the ablation of typical atrial flutter, reducing the surgical time and fluoroscopy time, and improve the success rate and safety of the procedure (41–44). For atrial tachycardia originating from the non-coronary cusp, the ICE catheter can be placed in the right atrium or right ventricular outflow tract to monitor the ablation process.

#### Application of intracardiac echocardiography in cryoablation

Pulmonary vein isolation (PVI) is the standard approach for treating atrial fibrillation, restoring and maintaining sinus rhythm (45). Cryoballoon ablation (CBA) has emerged as an established modality to perform PVI in patients with atrial fibrillation (45, 46). CBA is as effective as radiofrequency ablation in maintaining sinus rhythm, but CBA tends to be associated with more radiation exposure and higher contrast agent dose (45). In CBA for atrial fibrillation, the use of ICE to guide balloon positioning and assess closure can reduce the duration of X-ray exposure and the dose of contrast agent, without affecting the success rate and safety of the procedure (47). However, there is still a lack of data that ICE-guided CBA is clearly more efficient and safer than two-dimensional X-ray imaging-guided CBA in performing PVI. In addition, ICE-based color Doppler flow imaging (CDFI) requires less exposure to contrast agents. In PVI, the presence of flow around the balloon observed with CDFI indicates incomplete obstruction, in which case the operator should adjust the balloon position, without the need for venography (48). Therefore, ICE is an emerging option for those who cannot undergo fluoroscopy due to renal insufficiency or contrast media allergy.

**FIGURE 2:**
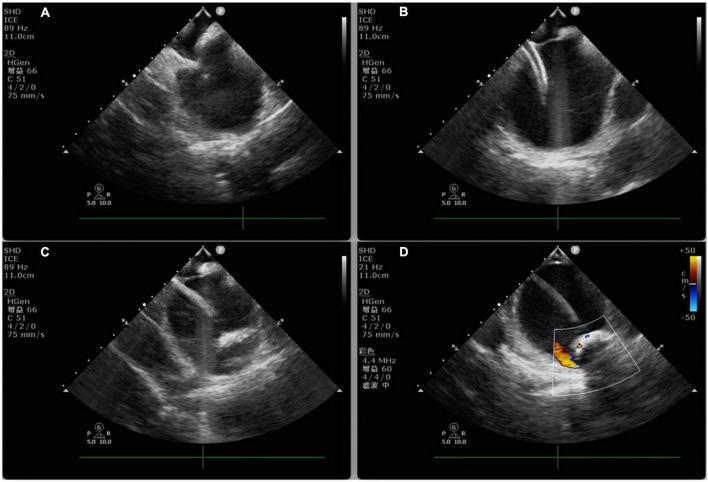
Intracardiac echocardiography-guided cryoablation. **(A)** ICE-guided low transseptal puncture; **(B)** cryoablation sheath delivered through the low transseptum; **(C)** cryoballoon delivered into the left superior pulmonary vein under ICE guidance; **(D)** ICE color Doppler ultrasound applied to assess cryoballoon closure.

Pulmonary vein isolation alone demonstrates a low success rate in patients with persistent atrial fibrillation. Given this, some studies reported the application of CBA for PVI with additional substrate ablation in the treatment of persistent atrial fibrillation in recent years: (1) left atrial roof linear ablation (49); (2) left atrial posterior wall isolation (PWI) (50); (3) segmental pulmonary vein isolation or extended pulmonary vein antrum ablation (51); (4) left atrial appendage isolation; (5) ablation of non-pulmonary vein triggers (52). The above studies suggest that the application of CBA for PVI with additional substrate ablation may improve the ablation success rate of persistent atrial fibrillation, but this finding remains to be confirmed by multicenter randomized controlled studies. PVI with additional substrate ablation mostly requires the application of non-balloon obstructive ablation techniques. In this case, imaging or blood flow monitoring by ICE may help to guide cryoballoon positioning and improve the efficiency and efficacy of ablation (53) (Figure 2). However, ICE-guided CBA should be performed by experienced and technically trained operators (Flow chart 3 shown in Supplementary materials).

#### Application of intracardiac echocardiography in reduction of catheter ablation-associated complications

Rapid diagnosis and prevention of potential complications during ablation is one of the most important functions of ICE (54). A recent study showed (55) an early mortality of 0.46% in patients undergoing catheter ablation for atrial fibrillation. Prompt management of postoperative complications and congestive heart failure may be crucial to reducing mortality. Although the experience of operators and their knowledge of indications are essential, immediate prevention, diagnosis, and management of surgical complications are particularly critical to reducing the mortality of atrial fibrillation ablation. Major complications associated with the leading causes of death include cardiac perforation during ablation, left atrial thrombus, esophageal injury, and pulmonary vein stenosis (55).

The application of ICE in catheter ablation can reduce the incidence of perioperative complications, especially the incidence of serious complications such as thromboembolism and cardiac tamponade (3, 55–64), and shorten the average length of stay of patients (60). Studies have demonstrated that ICE-guided circumferential pulmonary vein ablation with the Carto-Sound system is safe and feasible in patients with atrial fibrillation compared with conventional X-ray radiography (65), and there was no significant difference in the incidence of complications such as puncture site hematoma and cardiac tamponade between the two groups (65–67). The integration of ICE and electroanatomical mapping allows zero X-ray transseptal puncture and safe and effective ablation of left-sided tachycardia (including atrial fibrillation, atypical atrial flutter, left-sided accessory pathway, ventricular tachycardia, and focal atrial tachycardia) (68). In this way, the incidence of complications (stroke/transient ischemic attack, pericardial effusion, cardiac tamponade, pseudoaneurysm requiring surgery or intervention, esophageal injury, transient phrenic nerve palsy, and displacement of cardiac implantable device) within 30 days after surgery is 1.9%, and the incidence of transseptal puncture-related cardiac tamponade is 0.2%.

Acute cardiac tamponade is one of the most common serious complications associated with catheter manipulation during ablation. ICE can detect early pericardial effusion along the lower ventricular border and posterior left atrium, which can be managed by reversing anticoagulant therapy to prevent cardiac tamponade (54, 57, 59, 62, 63). Meanwhile, pericardiocentesis can be performed as early as possible, with a drainage tube placed if necessary. ICE also allows continuous monitoring of the dynamic changes of pericardial fluid during drainage (54, 57). The ICE catheter is advanced from the right atrium with the tip pointing anteriorly, and then deflected across the tricuspid valve and into the right ventricle where the inferoposterior border of the heart can be seen (54). Clockwise rotation at the interventricular septum reveals images of the left ventricular cavity, mitral valve, and posteroinferior space of the pericardium (54).

Thromboembolism is another serious complication associated with left cardiac ablation. ICE-guided catheter ablation allows real-time observation of the factors associated with increased stroke risk, such as thrombosis at the catheter, sheath, and endocardial lesion sites and clot formation on the ablation electrodes (54). Once a soft thrombus is detected by ICE, the clot can be aspirated into the sheath, and a higher dose of anticoagulant can be administered to prevent serious thromboembolic complications (54). If the thrombus is firmly attached to the catheter, ICE can guide to remove the thrombus into the right atrium (55).

Esophageal injury and atrio-esophageal fistula are important issues in atrial fibrillation ablation. The incidence of the atrio-esophageal fistula is 0.05–2%, while the esophageal injury is still common (54). The ability of ICE to identify the position of the esophagus in relation to the left atrium is comparable to that of magnetic resonance imaging. In addition, with the real-time imaging function of ICE, operators can monitor the position of the ablation catheter and the esophagus in real-time during catheter ablation, and reduce RF energy to reduce the risk of esophageal injury when the catheter ablates the area close to the posterior wall of the left atrium (54). However, there is still a lack of data from clinical studies on the effectiveness of ICE in monitoring the position of the esophagus during catheter ablation for atrial fibrillation (69).

Pulmonary vein stenosis is associated with ablation sites at the pulmonary vein antrum, likely when lesions are delivered in a more ostial location. This complication can be mitigated or avoided by accurately localizing the optimal ablation site with ICE (54, 61). ICE can also monitor development of tissue edema, a marker for energy delivery, at the ablation site. With ablation site real-time monitoring on ICE, energy delivery can be stopped immediately once manifestations of local overheating such as tissue blanching or microbubble generation are found at the ablation site, so as to prevent further damage, including risks of steam pop, and potential excessive ablation that may increase risks of PV stenosis (61, 70). Moreover, ICE can measure pulmonary vein flow velocity. In patients undergoing repeat ablation procedures, the application of ICE to measure pulmonary vein flow velocity in addition to assessing PV anatomy and vein caliber are important steps prior to re-ablation (54).

Complications associated with ICE application itself are rare. However, as the ICE catheter is relatively stiff and may result in vascular injury and/or perforation during its advancement, it should be advanced with care (57).

#### Short learning curve and low learning difficulty for intracardiac echocardiography-guided catheter ablation

**FIGURE 3:**
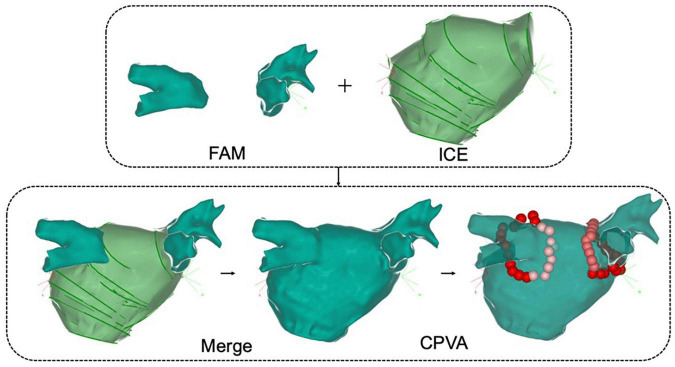
Intracardiac echocardiography combined with fast anatomical reconstruction for left atrial model reconstruction. FAM, fast anatomical mapping; Merge, ICE combined with three-dimensional reconstruction; CPVA, circumferential pulmonary vein ablation.

The ability to accurately and clearly delineate cardiac anatomy is directly related to procedural efficiency, efficacy, and safety. False lumens and other anatomic inaccuracies are inevitable with conventional contact-type three-dimensional reconstruction, affecting the operators’ judgment of ablation targets or special structures. ICE, as a non-contact three-dimensional reconstruction technique, is not limited by catheter position. With ICE, the atrial body, pulmonary veins, atrial appendages and other structures can be entirely reconstructed in an objective and accurate manner through simple sector adjustments. Furthermore, based on the integration of ICE and contact-type reconstruction of key structures, a more realistic and accurate anatomical model can be obtained after image fusion, creating an anatomic framework for subsequent ablation (Figure 3).

During ablation, ICE can not only track the ablation catheter in real time and identify the catheter position in complex structures such as the left atrial appendage-pulmonary vein ridge, but it can also help avoid esophageal injury and excessive ablation at thinner parts of the myocardium.

Intracardiac echocardiography-guided standardized approach to ablation procedures may improve several procedural outcomes. Beginners can obtain satisfactory ablation results with ICE-guided low X-ray catheter ablation for atrial fibrillation, with an average fluoroscopy time of 2.3 ± 3.0 min. The learning curve is short, with the fluoroscopy time dropping rapidly to 9 s from 3.8 min during a study evaluating learning curve (71).

Experts’ recommendation: (1) If possible, ICE-guided transseptal puncture is recommended, especially for patients with abnormal interatrial septal anatomy. (2) Routine TEE or left atrial and pulmonary vein CT examination should be performed before catheter ablation in patients with atrial fibrillation to rule out left atrial appendage thrombus and preliminarily assess the shape and size of the left atrial appendage; for patients with suspected thrombus that is difficult to distinguish preoperatively or unable to tolerate and unable to undergo left atrial and pulmonary vein CT or TEE examination, ICE can be applied intraoperatively to provide additional assessment for left atrial appendage thrombus and re-assess the shape and size of the left atrial appendage; meanwhile, ICE can also be used as an alternative monitoring and assessment technique in patients intolerable to TEE. (3) If possible, ICE-guided catheter ablation requiring low X-ray or zero X-ray is recommended for atrial fibrillation in medical centers to reduce the radiation exposure of patients and surgeons. (4) Imaging and blood flow monitoring by ICE may help to guide cryoballoon positioning and improve the efficiency and efficacy of ablation. (5) Catheter ablation for patients who are not suitable for radiation including pregnant women should be performed under ICE guidance. (6) During the training of new electrophysiologists, instruction in ICE is recommended.

### Application of intracardiac echocardiography in interventional diagnosis and treatment of ventricular arrhythmia

Intracardiac echocardiography plays an important role in the catheter ablation for ventricular arrhythmias, just as in interventions for atrial arrhythmias. It is mainly used to monitor cardiac structures in real-time, reduce the duration of surgery and radiation exposure (72), delineate dyskinetic areas in detail (73), and rapidly identify intraoperative complications (74).

#### Application of intracardiac echocardiography in fascicular ventricular tachycardia

Left posterior fascicular ventricular tachycardia (VT) is the most common type of idiopathic ventricular tachycardia, and its electrophysiological mechanism remains controversial. It is traditionally considered to be a macro-reentrant arrhythmia originating from the left posterior fascicle, which needs to be differentiated from the ventricular tachycardia originating from papillary muscles in clinical practice. However, definitive differentiation between the two based on ECG and EP is sometimes difficult. In fact, it has been proved that the mechanism of a part of fascicular ventricular tachycardia is closely related to such structures as Purkinje fibers and false tendons around the papillary muscles (75). For this part of left posterior fascicular ventricular tachycardia, the target for successful ablation may not be conventionally in the left mid-posterior septum, but around such anatomical structures as the left posterior papillary muscle and/or the false tendon attached to it. Since these structures and septa are very close to each other, especially during episodes of ventricular tachycardia when cardiac chambers shrink, and these structures are anatomically complex in three dimensions, they may be difficult to distinguish in an ordinary three-dimensional mapping system. By visualizing the position of the ablation catheter relative to the left interventricular septum, papillary muscles and false tendon intraoperatively, ICE can identify the true anatomical position of the optimal target, as well as the degree of contact between the catheter and the target, which is of great significance in further exploring the mechanism of left posterior fascicular ventricular tachycardia and improving the success rate of ablation. Therefore, in recent years, ICE has been gradually recognized for its advantages in the mapping and ablation of left posterior fascicular ventricular tachycardia.

#### Application of intracardiac echocardiography in ventricular arrhythmia originating from outflow tracts

Idiopathic outflow tract ventricular arrhythmias mainly include monomorphic ventricular premature beats, non-sustained ventricular tachycardia, and sustained monomorphic ventricular tachycardia. Right ventricular outflow tract ventricular arrhythmia is the most common type of ventricular arrhythmias in clinical practice, mostly idiopathic, accounting for about 80% of outflow tract ventricular arrhythmias (76). In recent years, with the further understanding of right ventricular outflow tract ablation, reversed U-curve ablation above the pulmonary valve has become a common approach to deliver the ablation catheter to the target site with adequate contact force and stability (77). However, without ICE-guided precise anatomical orientation of ablation targets, it remains difficult to determine the exact ideal ablation position: Whether the catheter tip is placed above the pulmonary valve? Is the catheter in place? The anatomical position is difficult to determine by angiography alone. However, ICE can clearly demonstrate the adjacent relationship between the ablation catheter and the pulmonary valve, pulmonary artery and right ventricular outflow tract, and observe the contact between the catheter and the corresponding anatomical position of the ablation target in real time. Moreover, ICE along with its three-dimensional model can help operators understand the anatomical sites of mapping and ablation in a more intuitive way, thus likely improving the success rate. A close adjacent relationship between the left coronary artery and the anteroseptal site of the right ventricular outflow tract can be confirmed by ICE combined with electroanatomical mapping. Continuous ICE images can be obtained by rotating the imaging catheter in the right ventricle to mark the anatomical images of the left coronary artery. To delineate the structure of the ventricular outflow tract, the ICE catheter is usually placed in the right atrium and rotated clockwise from the tricuspid valve, with the aortic valve on the long axis and the pulmonary valve on the short axis. In addition, placement of the ICE catheter directly within the RVOT can help to visualize that region.

The right ventricular outflow tract myocardial tissue anatomically extends to the pulmonary valve and pulmonary artery, making the positioning of ablation targets more complex. Some cases failing with subvalvular ablation may be successfully treated by supravalvular ablation. However, for supravalvular ablation, a transvalvular approach may lead to valve injury and other complications. In this case, the application of ICE not only helps to avoid such complications, but also enables sound reconstruction and real-time monitoring of the pulmonary artery, aortic valve, left anterior descending artery and right ventricular outflow tract during the surgery, and avoids the use of fluoroscopy and contrast agent and the occurrence of such complications as valvular insufficiency. With ICE placed in the right atrial appendage to obtain real-time cross-section images of pulmonary valves (78), it is easier to locate each pulmonary valve and determine the ablation target, thus performing a successful ablation.

Given the complex anatomy (coronary arteries, etc.) adjacent to the right ventricular outflow tract, RF energy transmitted close to the coronary arteries may cause obstruction of major epicardial vessels (e.g., left anterior descending artery) and possibly myocardial infarction. It is therefore essential to identify the anatomic location of these structures, traditionally using coronary angiography. ICE enables accurate reconstruction and real-time dynamic observation of proximal arterial anatomy during ablation, avoiding the use of contrast agents and further reducing risk to patients. Right ventricular outflow tract ventricular arrhythmias usually originate from the root of the pulmonary artery. Without ICE, operators may be unable to determine the relationship between the ablation target and a specific anatomical structure at that site due to trabecular muscles and fibrous tissues arranged in a crisscross pattern at this site, the low resolution of 3D navigation and the 2D image overlay of fluoroscopy/radiography. However, ICE can visualize fine anatomical landmarks of the heart in real time and guide the catheter operation (17) throughout the procedure, thereby increasing operators’ confidence and shortening their learning curve. Therefore, ICE is expected to improve the success rates and reduce complications for these procedures.

**FIGURE 4:**
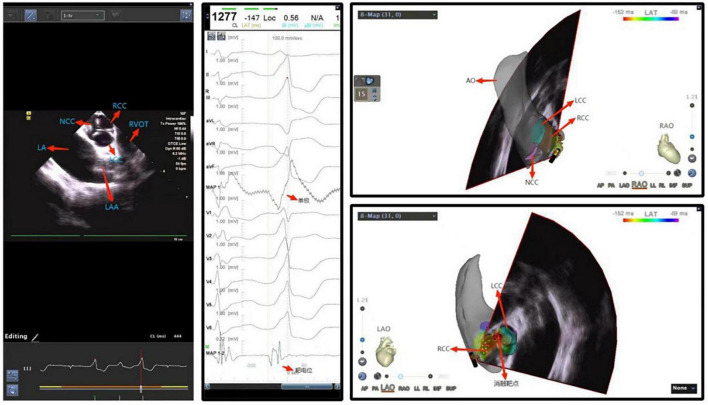
Ventricular premature beat between left and right coronary cusps. NCC, non-coronary cusp; RCC, right coronary cusp; LCC, left coronary cusp; LA, left atrium; LAA, left atrial appendage.

With improved understanding and experience, the recognized incidence of ventricular arrhythmia originating from the left ventricular outflow tract is increasing year by year, especially ventricular premature beat/ventricular tachycardia originating from the aortic sinus and its adjacent areas. As the aortic sinus is located in the central part of the heart and with adjacent tissues are critical and anatomically complex, patients whose ventricular arrhythmias originate from these anatomically complex regions (coronary artery, etc. Figure 4), may be at risk for serious complications such as valve injury, cardiac perforation, even acute myocardial infarction and complete atrioventricular block. As the aortic root is the continuation of the left ventricular outflow tract, where the blood flow is fast and under high pressure, the ablation catheter may not fit easily and stably, and sometimes ablation energy can be difficult to deliver effectively. Therefore, anatomic reconstruction of the area around this ablation target is particularly important. ICE can construct a three-dimensional model of the left ventricular outflow tract, assess the distance between the artery and the catheter, eliminate the potential risks of ablation within this area, and clarify the feasibility of ablation. With the ICE probe placed in the right atrium to image anteriorly, the mapping position and the position of the ablation catheter in relation to the aorta, aortic valve, coronary ostium and other structures can be monitored in real time, which helps to reduce the potential damage to the aortic valve or coronary artery, increase surgical safety and improve the success rate of ablation. In particular, ICE also plays an increasingly important role in the ablation of pediatric outflow tract ventricular arrhythmias (79).

#### Application of intracardiac echocardiography in ventricular arrhythmia originating from papillary muscles

**FIGURE 5:**
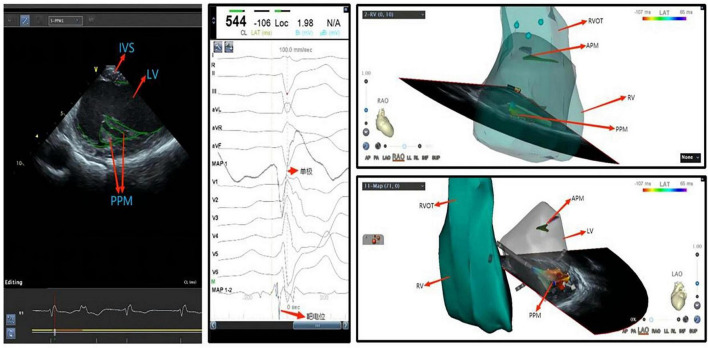
Ventricular premature beat of left ventricular posterior papillary muscle. IVS, interventricular septum; LV, left ventricle; APM, anterior papillary muscle; PVOT, right ventricular outflow tract; PPM, posterior papillary muscle; RV, right ventricle; LV, left ventricle.

**FIGURE 6:**
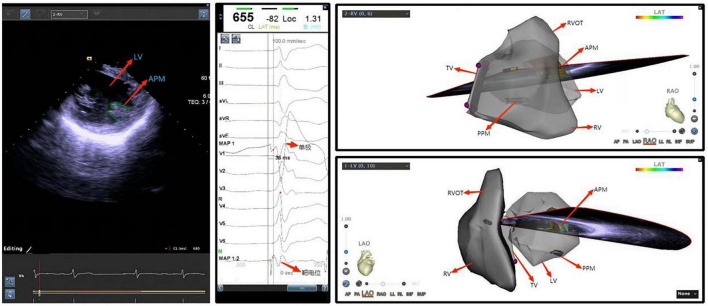
Ventricular premature beat of left ventricular anterior papillary muscle. LV, left ventricle; APM, anterior papillary muscle; TV, tricuspid valve; PVOT, right ventricular outflow tract; PPM, posterior papillary muscle; RV, right ventricle; LV, left ventricle.

Ventricular arrhythmias originating from the left and right ventricular papillary muscles and moderator bands are relatively common in clinical practice. As these anatomical structures are located on the inner surface of the cardiac chamber, neither conventional X-ray radiography nor three-dimensional imaging systems can visualize their locations. In addition, these structures are not always in a fixed position due to the catheter advancement as they have smooth surfaces and move independently during the cardiac cycle. In this case, in the mapping and ablation for such arrhythmias with conventional approaches, the catheter may be difficult to direct toward the ablation target or remain stable during ablation (Figure 5). In particular, the contact of the catheter to the left ventricular anterior papillary muscle is challenging when the electrophysiologist only applies X-ray for imaging guidance, while ICE shows great advantages (Figure 6). As a result, ablation for such arrhythmias may have lower success rates and a higher recurrence rate, compared with that for ventricular arrhythmias originating from other sites (80). In recent years, ICE has become an indispensable adjunct to such arrhythmias in the following ways: (1) to visualize the anatomical location of papillary muscles and quickly guide the preliminary placement of mapping catheter; (2) help the surgeon clarify the segments (tip, middle, and root) and sides of the papillary muscle where the catheter is located through real-time ultrasonic sector and three-dimensional tracking, so as to determine the exact position of the optimal target; (3) ensure good contact between the catheter and the papillary muscle under real-time monitoring and through subtle adjustment of the catheter, contributing to accurate mapping (pacing and activation mapping) and effective ablation; (4) help to observe the ablation effect and degree of injury (tissue edema or blanching) during surgery, monitor the occurrence of pop and complications, reduce radiation exposure and improve safety (Flow chart 4 shown in Supplementary materials).

#### Application of intracardiac echocardiography in ventricular arrhythmias originating from the top of left ventricle (aka left ventricle summit) and other special sites in cardiac chambers

**FIGURE 7:**
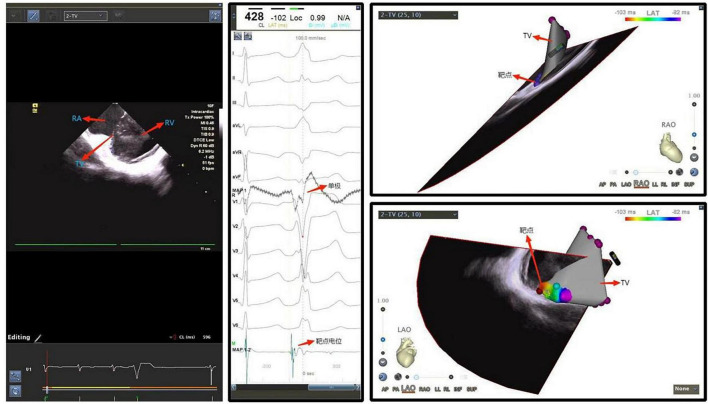
Ventricular premature beat originating from the tricuspid annulus. RA, right atrium; TV, tricuspid valve; RV, right ventricle.

Ventricular arrhythmias may originate at the top of the left ventricle, or LV summit, often the mid-myocardium or epicardium, located at the junction between the aorta and the left ventricular inflow tract. In this junction also lies a layer of tough fibrous tissue on the intimal surface that connects the aorta and mitral valve, often termed the aorto-mitral continuity, with the epicardial surface is close to the coronary artery and covered with a thick layer of epicardial adipose (81). Catheter ablation for arrhythmias arising from this region comes with a low success rate in this area because the proximal coronary artery is covered with a thick layer of epicardial adipose, and catheter ablation in this area may pose a potential risk of injury to these vessels (82). Coronary sinus or large cardiac vena cava venography is usually used to guide positioning in clinical practice, and often endocardial ablation as well as epicardial ablation are adopted for treatment. The application of ICE assisted catheter ablation has demonstrated that: Although ICE has resolution that is too low to delineate distal small vessels, when the catheter is placed in the right ventricular outflow tract, it still can delineate the left anterior descending coronary artery, the left ventricle, the aorta and other anatomical structures, presenting the 3D space position and adjacent relationship to guide the ablation catheter accurately in this challenging anatomy. The operator can then perform ablation successfully in this area in the absence of venography or angiography (83). ICE also plays a unique role in cardiac chambers that are difficult to be visualized by X-ray imaging guidance and in special types of ventricular premature beats that are difficult to be located by surface ECG (Figure 7).

#### Application of intracardiac echocardiography in ventricular tachycardia with ischemic cardiomyopathy

**FIGURE 8:**
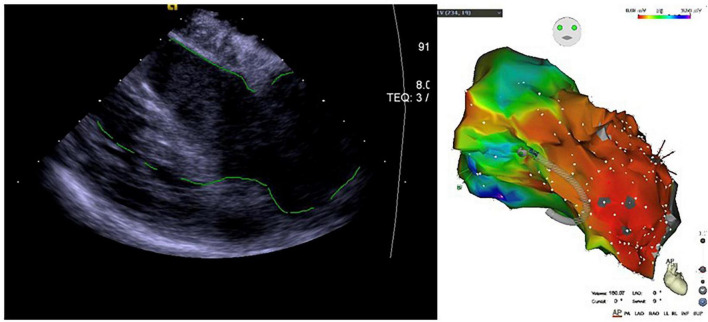
Application of ICE in ischemic cardiomyopathy with ventricular aneurysm-induced ventricular tachycardia.

In ischemic cardiomyopathy, ventricular scar may mediate macroreentrant ventricular tachycardia. Some patients after infarct may eventually develop ventricular aneurysm, further complicating the anatomic considerations for catheter ablation. The ablation strategy for these patients is most commonly guided by either activation mapping and/or substrate mapping in the scar area. Ablation in ventricular tachycardia patients with ischemic cardiomyopathy is challenging in part because cardiomegaly and often thinned ventricular walls may increase the challenge and risks to mapping and ablation. Preoperatively, scar regions can be approximately localized by transthoracic echocardiography (TTE) and cardiac CT/MRI. Contours of the left (or right) ventricle and associated structures can be reconstructed with ICE via the right ventricle or sometimes the right atrium or CS. ICE can localize the catheter in relationship with the ventricular tissues, resulting in likely lowered risk of cardiac perforation due to excessive contact force. Especially in ischemic cardiomyopathy patients with ventricular aneurysm, left ventricular anatomy is often distorted, so that ICE reconstruction of a ventricular aneurysm and the aneurysmal neck can be helpful to define the anatomy and hence potential ablation target (84) (Figure 8).

Another important advantage of ICE for such patients is the delineation and quantification of the scar area. The scar area appears as a hyperechoic area on the ultrasound sector, and the marginal area appears as a mixture of medium to high echo densities, which is significantly different from that of normal ventricular myocardium (85). A study of 18 patients with organic ventricular tachycardia (83% with ischemic cardiomyopathy) showed that the ICE-defined scar area was 86% concordant with the scar area measured by substrate mapping (86). This approach is not limited to ischemic cardiomyopathy (87). A propensity score study that ultimately included 1324 patients with organic ventricular tachycardia showed a lower readmission rate and reoperation rate for ventricular tachycardia in the ICE group than those in the non-ICE group (88).

Experts’ recommendation: (1) As a safe, effective, efficient, and comprehensive approach with unique advantages in identifying and locating ablation targets and specific cardiac anatomy, and with superiority to other techniques in identifying small abnormal foci, ICE is recommended as an imaging modality in radiofrequency ablation of left and right ventricular outflow tract arrhythmias, post-TAVR ventricular tachycardia and VSD-induced ventricular tachycardia in adults. Radiofrequency ablation of ventricular tachycardia augmented by ICE may also reduce readmission rates, the possibility of repeated ablation, and the incidence of complications. (2) ICE can be actively applied in ventricular arrhythmia patients with ventricular wall dysfunction who require ablation.

## Application of intracardiac echocardiography in congenital heart disease

### Application of intracardiac echocardiography in atrial septal defect and patent foramen ovale

Intracardiac echocardiography is now the most widely used technique in ASD and PFO closure among the interventional closure treatments for various congenital heart diseases. Statistical results in the United States indicate that the use of ICE in ASD closure has increased from an initial 9.7% to more than 50% today (89). In the past, TEE was considered the gold standard to guide interventional closures of ASD and PFO (90, 91), but now several studies have confirmed that use of ICE has better safety and clinical outcomes than TEE and is a more suitable ultrasonographic approach to guide the closure of secondary ASD and PFO (92, 93).

**FIGURE 9:**
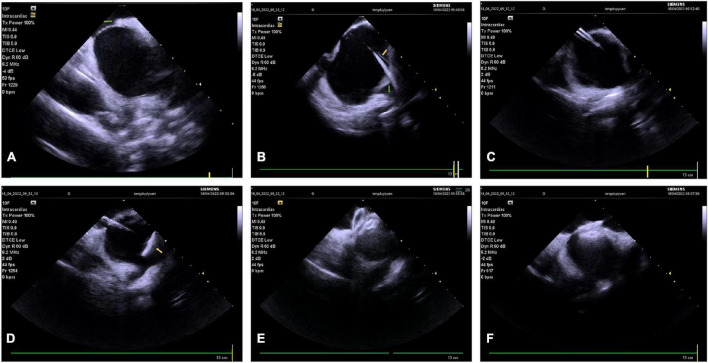
Patent foramen ovale closure procedure guided by ICE. **(A)** PFO slit (green arrow). **(B)** The guidewire passes through the PFO slit (yellow arrow points to the highlighted echo which represents the guidewire, and green arrow points to the left superior pulmonary vein). **(C)** Send the delivery sheath to the ostium of the left superior pulmonary vein. **(D)** Release the left plate of the closure device (the yellow arrow points to the left plate). **(E)** Perform a pull test after the closure device is fully expanded. **(F)** The closure device is released. PFO, patent foramen ovale.

Intracardiac echocardiography has a higher image resolution than TEE. Despite the lack of multiplanar imaging capabilities, it can still image the interatrial septum from multiple views with its flexible probe, thereby obtaining images similar to or better than those obtained by TEE (94). ICE can accurately assess the dimensions of the fossa ovalis, the diameter of interatrial septum, the width or length of tunnel, and the diameter of tunnel inlet and outlet; it can also display any shunt at the atrial level in patients with PFO and determine whether there is a long valvula venae cavae inferioris or Chiari’s network, interatrial septum aneurysm, double-layer septum and other abnormalities and special complex structures (95). In ASD closure, ICE can accurately measure the diameter of the ASD on multiple views before and after closure device release, assess the length and thickness of ASD edges, such as superior and inferior vena cava edges, anterosuperior edge and superior edge of interatrial septum, and posterior part of diaphragm, and display the relationship between ASD and surrounding structures (right pulmonary vein, coronary sinus, mitral valve, tricuspid valve, etc.), which is helpful in selecting an appropriate size of closure device and to rule out the possibility of other defects or rare conditions such as venous sinus ASD (96). Moreover, real-time color Doppler flow monitoring by ICE can be performed intraoperatively to further exclude other potential defects. ICE can better display the posterior and inferior edges of interatrial septum (97), as well as the relationship between closure device and superior vena cava (especially in young children) than TEE (98). It can be used in the closure of complex ASDs such as ASD with diameter of more than 38 mm and/or ASD with edge damage except anterosuperior edge damage, porous ASD and ASD with impaired systolic function (99–101). In addition, ICE has been shown to be more accurate than TEE in performing anatomical measurements and guiding implantation (particularly for patients with smaller left atria) (98). Intraoperatively, ICE can monitor and guide the operation process in real time in an effective and safe manner, assist the surgeon in accurately locating the PFO slit, and make the guidewire pass through the slit quickly, thereby shortening the operation time; guide the surgeon to release the closure device under direct vision throughout the procedure, determine whether the closure device is tilted or in a wrong position, and observe whether the closure device is stable, avoiding the X-ray artifacts caused by TEE transesophagel probe. In addition, the surgeon can confirm whether there is residual shunt by injecting normal saline and/or by color Doppler examination under the guidance of ICE. In PFO closure, in case of a particularly long tunnel, the closure may be performed by transseptal puncture, otherwise, there may be a large amount of residual shunt (102). ICE is considered an important tool to guide the transseptal puncture (103) (Figure 9).

Another significant advantage of ICE is that it can significantly shorten intraoperative X-ray exposure time (104), effectively reducing the radiation hazard to patients (especially children, pregnant women, obese patients) and surgeons. It also does not have major drawbacks that are associated with TEE, such as esophageal perforation, the need for general anesthesia/deep sedation, and possible associated complications from endotracheal intubation (Flow chart 5 shown in Supplementary materials).

Cost is one of the major factors restricting the wide application of ICE in clinical practice. ICE itself is more expensive than TEE, but if ICE is used, general anesthesia may be exempted and the average hospital stay can be shortened (89, 105), thus reducing other costs during the hospital stay.

### Application of intracardiac echocardiography in other common congenital heart diseases

Currently, there is relatively little experience with ICE for PDA and VSD procedures. Percutaneous interventional PDA closure is currently the standard of care for PDA, and conventional therapy is aortography-guided interventional closure. However, for most patients with PDA, a large amount of contrast agent is required in aortography, and in some cases, images obtained are not clear enough to assess PDA anatomy (106, 107), so the size of PDA may be underestimated, thus affecting the surgeon’s choice of device, and leading to risk of embolization (108). In addition, patients with contrast agent allergy or renal insufficiency (108–110) are at risk with aortography. The results of available studies suggest that ICE is comparable to aortography or cardiac CTA in terms of the accuracy of measuring PDA diameter on the pulmonary artery side, but the amount of contrast agent required for ICE-guided PDA assessment is significantly lower than that for aortography. Therefore, ICE is now considered a reasonable substitute for aortography as a routine test for assessing the relevant structures of PDA, especially in patients with large PDA, renal insufficiency, or contrast agent allergy (111).

In the percutaneous closure of membranous VSD, TEE plays an important role in the anatomical assessment of the defect and the surgeon’s intraoperative decision. However, due to the long duration of VSD closure, TEE related examinations should be performed under general anesthesia. ICE provides clear images of the membrane of VSD, and its measurement results are similar to those obtained by TEE; in addition, ICE and TEE are comparable in judging the relationship between the defect and the aortic valve and tricuspid valve, measuring the size of the defect and giving guidance at different stages.

Experts’ recommendation: (1) ICE performs well in anatomical measurements of relevant structures, real-time guidance of closure device implantation, assessment of post-closure residual shunt, etc. If possible, medical centers should apply ICE to guide the interventional procedure of secondary ASD and PFO; (2) ICE is recommended to guide the interventional closure in patients with complex or special ASD and PFO, especially the closure in patients with complex ASD, intolerant to TEE, with impaired left ventricular systolic function and unable to receive fluoroscopy, especially children, pregnant women, obese patients; (3) ICE is recommended to guide the interventional closure of PDA in patients with large PDA, renal insufficiency, and contrast agent allergy; (4) ICE is recommended to guide the closure in VSD patients with complex anatomy.

## Application of intracardiac echocardiography in interventional procedure of valvular heart diseases

### Transcatheter aortic valve replacement interventional procedure

Severe symptomatic aortic valve stenosis is a life-threatening disease with a 2-year mortality rate as high as 50% (112), and TAVR has become an effective treatment for this disease (113, 114). However, there are many risks associated with TAVR, including annular rupture, ventricular perforation, aortic dissection, coronary occlusion, and valve prosthesis displacement, as well as prosthetic valve PVL due to improper valve placement. Most complications cannot be detected in the early stages without guidance from echocardiography (115). Preoperative and perioperative imaging is essential for accurate determination of valve size, and assessment of postoperative aortic and paravalvular regurgitation, and other complications (116). TEE is currently commonly used for intraoperative guidance to TAVR in China and elsewhere. As an alternative to TEE, ICE dispenses with general anesthesia and endotracheal intubation during TAVR, especially in patients with esophageal disease.

Kadakia reported a TAVR case (117) that was successfully treated under the guidance of 3D ICE imaging. We found that the ICE was comparable to conventional TEE imaging in evaluating the valve position and aortic incompetence during TAVR and demonstrated comparable diagnostic imaging quality to multidetector computed tomography (MDCT) (118). Given this case report, ICE-guided TAVR may become an important alternative to TEE imaging and may allow for low-intensity sedation or anesthesia, potentially improving procedural safety and logistics (117). The aortic annulus and aortic sinus diameters measured by ICE were found to be comparable to those obtained by MDCT (116). Additional studies have assessed the intraoperative guidance of ICE and TEE during TAVR (119), where 50 patients with severe aortic valve stenosis scheduled for TAVR were randomized into two groups for ICE monitoring and TEE monitoring, respectively. The results showed that ICE was indeed capable of continuous monitoring. The ICE group had a much lower need for probe repositioning during the procedure. The ICE view displayed a higher coaxiality with the ascending aorta, indicated by the length of the ascending aorta depicted. In ICE group, both coronary ostia were visualized more frequently. The annulus measurements by ICE correlated closely with the readings by conventional TEE.

Intraoperative pressure gradients were underestimated by TEE compared with conventional measurements, but not by ICE. Both ICE and TEE detected new intracardiac thrombi. In this study, the authors concluded that ICE was compatible with sedation and local anesthesia and could be used for guidance in place of TEE. Also, it appeared to better match the operational flow during TAVR than TEE. In a study of 21 patients scheduled for TAVR, the major intraoperative imaging modality was 3D ICE (118). These patients were selected unanimously by the multidisciplinary TAVR team. This study is the first to prospectively evaluate the safety and feasibility of ICE-guided TAVR in the absence of endotracheal intubation. It is concluded that ICE is safe and feasible in selected patients in the absence of major complications, and intraoperative ICE can detect perivalvular leak and help guide necessary treatment. With the continued emphasis on the use of conscious sedation in TAVR procedures, it is very important to consider ICE as the major intraoperative imaging tool. 3D ICE probes are currently available for volumetric imaging; however, measurement of cross-sectional size of annular valves is difficult in its present form (105, 120). In addition, ICE makes it easier to measure tricuspid regurgitation (TR) and aortic valve flow velocity and to assess perioperative pulmonary arterial pressure and other hemodynamics (121). ICE can measure the aortic valve complex and provide more precise aortic pressure gradients (119, 121). In addition to providing imaging guidance, ICE dispenses with endotracheal intubation, shortens the operation time, and avoids the complications induced by general anesthesia and TEE (122). Therefore, ICE-guided TF TAVR in the absence of endotracheal intubation is a viable option in patients deemed appropriate by the multidisciplinary team.

Para-valvular leak is one of the most common complications of TAVR. A case of PVL on postoperative echocardiography and cardiovascular imaging was described (114). Eccentric aortic regurgitation after TAVR monitored by TEE is easily misdiagnosed as PVL, while ICE can accurately evaluate the main complications during the procedure, provide more accurate images, and further evaluate the cause and severity of regurgitation (114, 121). In this patient, the diagnosis of PVL was confirmed by ICE, and the eccentric aortic regurgitation was caused by the frozen tip of bioprosthetic valve of TAVR. Therefore, this study concluded that ICE is a reasonable alternative to or at least a complement to standard imaging modalities for assessment after TAVR implantation.

As for the operation of the ICE catheter during the TAVR procedure, one should advance the 8-Fr AcuNav™ catheter to the superior vena cava from the right internal jugular vein and rotate the catheter counterclockwise to obtain images of the ascending aorta and assess its anatomy preoperatively. Images of the interatrial septum can be obtained when the catheter is advanced a few centimeters further to judge whether there is ASD or PFO. Counterclockwise rotation is continued to obtain long-axis views of the right atrium, tricuspid valve, and right ventricle, where the preoperative TR and estimated right ventricular systolic pressure can be measured. When the catheter is advanced counterclockwise with forward flexion in the right ventricle, the long-axis view of the left ventricle displays left ventricular contraction and pericardial effusion, and this position can be maintained during much of the operation. When the catheter is returned clockwise and pulled to the right atrium, the long-axis view of the aortic valve is obtained, where the preoperative aortic valve velocity and the diameter of the aortic valve complex can be measured. After TAVR, evaluation for complications such as PVL from the margin of the non-coronary valve leaflet can be performed (121).

There are limitations associated with ICE, including the need for additional venous access, the learning curve related to new devices, and the possible increased cost (115). Special care is required in the operation of the ICE probe to avoid arrhythmias and perforation of the right heart and vena cava (123).

Experts’ recommendations: (1) ICE is a recommended alternative to TEE for TAVR in elderly patients with aortic valve stenosis who have esophageal lesions or are not suitable for general anesthesia; (2) ICE is equivalent to CT 3D reconstruction in the measurement of aortic valve and supravalvular and subvalvular structures and detection of possible complications during and after operation, and even superior to TEE in some cases; (3) It is suitable for surgeons experienced in both TAVR and ICE (Flow chart 6 shown in Supplementary materials).

### Transcatheter mitral valve intervention

Intracardiac echocardiography-guided mitral intervention includes Balloon Mitral Valvuloplasty, Transcatheter Edge-to-Edge Repair (TEER), and Transcatheter Mitral Valve Replacement (TMVR). ICE-guided transseptal puncture, ICE catheter access to the left atrium, and imaging and functional assessment of the mitral valve are fundamental operations of these therapeutic approaches, and we will uniformly describe them in detail. Additional procedures with different approaches are described in the corresponding sections.

#### Transseptal puncture

Transseptal puncture is a key step in mitral valve intervention, and it directly affects the success rate of mitral valve intervention. Under fluoroscopic guidance, deliver the ICE probe to the inferior position of the right atrium via the femoral vein, i.e., at the level of the tricuspid annulus, and appropriately rotate the catheter, until the right atrium, tricuspid valve, right ventricle, and right ventricular outflow tract can be visualized (home view). At this point, the posterior leaflet of the tricuspid valve is usually in the direction of 9 o’clock, while the anterior leaflet (or septal leaflet) is in the direction of 3 o’clock. This is the most fundamental view of ICE. Under fluoroscopy, deliver the ICE probe to the middle of the right atrium, rotate the catheter in the clockwise direction with a slight P curve to obtain the long-axis view of the interatrial septum, and along with the interatrial septum, its junction with the superior and inferior vena cava can be visualized. Further rotate the catheter in the clockwise direction with P curve to obtain the short-axis view of the atrial septum. At this point, the anterior (aortic) and posterior borders of the interatrial septum can now be visualized. Once the sheath/needle drag procedure is started, the “Tenting sign” of the puncture needle can be identified in the long-axis view of the interatrial septum. Then, rotate the ICE catheter in the counterclockwise direction until both the needle tent and the mitral annulus can be visualized to determine the optimal level of the puncture site.

#### Intracardiac echocardiography catheter access to the left atrium

After successful puncture, fix the puncture needle, push the dilator sheath into the left atrium, withdraw the sheath core, and the “tram track sign” can be observed in the ultrasound image. Deliver the stiffened guidewire to the left superior pulmonary vein or place in the left atrium, preferably looped for stability and ability to track the sheath. At this point, it should be determined that the activated clotting time (ACT) is within the therapeutic range before subsequent operations. The peripheral arterial balloon dilates the interatrial septum so that the sheath can smoothly pass through the interatrial septum. Adjust the A/P knob of the ICE catheter under fluoroscopy to align the catheter with the track of the stiffened guidewire, and gently push the catheter into the left atrium. In case of resistance during the procedure, slightly adjust the R/L knob or rotate the catheter in the clockwise/counterclockwise direction and push the catheter again, or adjust the ICE catheter under left or right anterior oblique fluoroscopy.

#### Imaging of the left heart system

After entering the left atrium, the ICE ultrasound probe can display images of the left atrial appendage, pulmonary veins, and mitral valve. Preoperative TEE for mitral valve intervention can identify most intra-atrial appendage thrombi, but when it is difficult to differentiate intra-atrial thrombi from normal pectinate muscle tissues by TEE, intraoperative application of ICE may be considered to re-identify the presence of intra-atrial appendage thrombi (31, 124). Release the tension knob, restore A/P and L/R curve to the middle position, rotate the ICE catheter until it faces the right shoulder, adjust the A/P curve to display the pulmonary veins, and obtain the pulmonary vein blood flow spectrum. In the middle position, rotate the ICE catheter until the mitral valve structure is visualized, slightly adjust the R/L curve to obtain the best bijunctional view, and assess the structure and function of mitral valve by color Doppler and multiplanar imaging.

#### Intervention for mitral incompetence

Henning et al. first reported the application of ICE in TEER (125). However, due to the lack of multiplanar 3D imaging technique and lack of experience in the application of ICE at that time, ICE was only used as an auxiliary imaging technique for TEE in TEER. Then, the team reported an additional case of TEER guided by ICE alone, in which they placed an ICE probe in the left and right atria of the patient, respectively, so as to simulate orthogonal 2D images (126). 4D ICE can perform real-time volumetric imaging and multiplanar reconstruction and may be an effective alternative to TEE for TEER intraoperative imaging in patients who cannot tolerate or have contraindications to TEE (127, 128). The steerable guide catheter (SGC) crosses the interatrial septum to the left atrium via a stiffened guidewire. A single transseptal puncture is recommended, with the SGC and ICE catheter entering the left atrium through the same puncture site. The location and extent of mitral regurgitation can be identified through the combination of ICE orthogonal 2D and 3D imaging and color Doppler. As 4D ICE can acquire 4D images and achieve multiplanar imaging, the ICE catheter can be fixed after the ICE probe acquires a mitral valve image in the left atrium, with only modest adjustments. The catheter delivery system (CDS) should be manipulated under continuous monitoring by ICE to avoid penetration of the CDS tip into the atrial sidewall. Rotate the ICE catheter in the counterclockwise direction after the CDS moves in the M direction and rotate the ICE catheter in the clockwise direction after the CDS moves in the P direction. Slowly and repeatedly adjust until the CDS is manipulated from the top of the left atrium to the central position of the mitral valve after completion of M-direction movement, with the tip of the clip in the annular plane above the center of the mitral valve. Under the guidance of multiplanar reconstruction and color Doppler, place the clip in the area with the most severe regurgitation. Under the guidance of orthogonal 2D and real-time 3D atrial images, make the clip arm perpendicular to the binding plane of the mitral valve, open the clip arm and place it into the left ventricle below the mitral leaflet. Clamp and release under continuous monitoring. Measure the position and extent of mitral regurgitation, transvalvular pressure gradient of mitral valve and pulmonary vein blood flow spectrum again after the operation, and compare with those before the operation. Perform supplementary clamping if necessary.

#### Intervention for mitral stenosis

Salem et al. first reported ICE-guided PBMV (129), and there are also more subsequent series of case reports on ICE-guided PBMV (130, 131). ICE guides intraoperative transseptal puncture, balloon positioning, evaluation of therapeutic effect, and monitoring of complications. The hemodynamic data measured by ICE are comparable to those measured by TTE and cardiac catheter. However, PBMV can be performed under local anesthesia combined with TTE monitoring in most patients, so ICE does not show significant advantages in terms of application in PBMV.

#### Transcatheter mitral valve replacement

Transcatheter mitral valve replacement guided by CS combined with ICE can be used to treat severe mitral incompetence caused by biological valve deterioration or prosthetic valve ring dysfunction and severe mitral annular calcification (MAC) (132, 133). ICE can guide intraoperative transseptal puncture, mitral valve crossing, valve positioning release, and functional assessment after valve release.

Due to the distance limitation of ICE imaging, the guidewire in the left atrium should be retained after mitral valve intervention, so that ICE probe can enter the left atrium for evaluation of therapeutic effect. Although ICE has shown many advantages in mitral valve intervention, it is not recommended to completely replace TEE at present due to the lack experience in relevant application, the lack of uniform operating specifications, the lack of popularity of 3D ICE and other reasons.

Experts’ recommendation: (1) ICE imaging from within the left atrium can be helpful if not critical in guiding transseptal puncture and evaluating the mitral regurgitation in the mitral valve intervention; (2) However, mitral valve intervention requires a large sheath to operate across the interatrial septum, which will affect the entry of the ICE catheter into the left atrium, and the treatment of mitral valve disorders mostly requires the guidance of 3D images, so it is currently not the main recommended method.

### Pulmonary valve intervention

Intracardiac echocardiography can clearly show the right ventricular outflow tract, pulmonary valve, and proximal pulmonary artery. Therefore, ICE comes with a good application prospect in the transcatheter intervention of pulmonary valve. At present, there is still little experience in the application of ICE in pulmonary valve intervention, limited to guiding transcatheter pulmonary valve replacement (TPVR) (134, 135). In the middle view of the right atrium, ICE can be used to evaluate tricuspid valve function and estimate right ventricular pressure (if TR is present). In the view of the right ventricular outflow tract, in addition to displaying the anatomical structure of the outflow tract, color Doppler and continuous Doppler can also be used to evaluate the valve regurgitation and transvalvular pressure gradient before and after the operation. ICE can also be used to monitor complications (such as pericardial effusion or thrombosis) during the operation.

Post-TPVR infective endocarditis (IE) is a potentially fatal complication. Previous studies showed that the incidence of post-TPVR IE and transcatheter pulmonary valve-related IE was 5.1 and 1.9%, respectively (136). TEE is the most commonly used imaging method to detect valvular vegetations and diagnose IE. However, due to the long distance of the pulmonary valve from the esophageal ultrasound probe, some lesions still cannot be detected by TEE. For cases in which post-TPVR IE is suspected but the test result by TEE is negative, ICE can assist in the definitive diagnosis (136, 137).

Experts’ recommendation: (1) ICE plays a good role in the assessment of pulmonary valve intervention and complications, and is not inferior to TEE; (2) Pulmonary valve intervention and ICE share the same approach and may interfere with each other; (3) It can be recommended as an effective alternative to TTE and TEE (Flow chart 7 shown in Supplementary materials).

### Tricuspid valve intervention

The incidence of TR is high in the elderly population (138). The one-year survival rate for patients with severe TR is 64% only (139). The effectiveness of drug therapy for TR is limited, and the mortality rate of surgical procedures is high (140). Therefore, various transcatheter treatment techniques have emerged in recent years, including transcatheter edge-to-edge repair, annuloplasty, and valve replacement. TEE is the standard imaging technique for tricuspid valve intervention, but there are also some technical problems, for example, the tricuspid annulus is far away from the esophageal ultrasound probe; the calcification of left heart valve prosthesis and tissues will interfere with the imaging; the delivery system and other devices will form acoustic shadows under ultrasound. Studies have found that the tricuspid valve structure is not adequately visualized in 50% of cases using TEE imaging alone in tricuspid valve intervention, and clips can be implanted under the guidance of ICE in 2/3 of these cases (141). Therefore, ICE is an important complementary technique to TEE in the intraoperative imaging of tricuspid valve intervention.

Due to the complexity and variability of the anatomical structure of tricuspid valve, there is no uniform standard for ICE imaging planes for tricuspid valve intervention. Hagemeyer et al. summarized the basic imaging planes of ICE in tricuspid valve intervention (142). In the home view, bend the ultrasound probe forward toward the right atrial free wall (left/right knob), and slightly rotate the catheter in a clockwise or counterclockwise direction. This view is often perpendicular to the long axis of the right ventricle, and the position of the clip arm can be determined based on the relationship with the valve leaflet. It is the ideal view for capturing the valve. To clearly visualize the anteroseptal junction, advance the catheter to a high level in the right atrium; to clearly visualize the posteroseptal junction, withdraw the catheter. Maintaining a stable field of view for intracardiac operation during intervention is essential and requires a second surgeon to assist in fixation or fine-tuning during operation to obtain an optimal imaging plane. 4D volumetric imaging ICE can bring great changes in tricuspid valve intervention. 4D volumetric imaging ICE is superior to TEE for visualization of the tricuspid annulus, especially the lateral annulus, and can be used for transcatheter annuloplasty, valve repair, and valve replacement for tricuspid in competence (140, 143). It should be noted that intravenous anesthetic drugs reduce systemic pressure while mechanical ventilation increases intrathoracic pressure. These will affect the accurate assessment of TR during operation. The application of the ICE system in conscious TR patients under local anesthesia can avoid these effects, making the assessment of TR more accurate and reliable.

Experts’ recommendation: (1) The image of ICE in transcatheter tricuspid valvuloplasty is non-inferior to, or potentially superior to that of TEE; (2) It is recommended that patients with throat or esophageal lesions and anesthesia contraindications should receive the guidance of ICE; (3) The guidance of 3D/4D ICE will have a great role in promoting the tricuspid valve intervention (Flow chart 8 shown in Supplementary materials).

### Paravalvular leak intervention

Long-term follow-up after surgical heart valve replacement has found that PVL occurs in 5–17% of patients (144, 145), and the incidence of PVL is three times higher with TMVR than with TAVR (146). PVL is also an important complication of TAVR, and it is mostly seen in early self-expanding valves (147, 148). Intervention is required when patients with moderate to severe PVL present with congestive heart failure and/or hemolysis. Surgical repair or valve replacement is the main intervention with high mortality. As interventional techniques and closure devices continuously develop, a growing number of centers use percutaneous PVL closure. 2020ACC/AHA guidelines state that percutaneous closure is recommended for PVL patients with high risk or contraindications for surgery, NYHA grade III/IV or refractory hemolysis, and appropriate anatomical structure (class IIa, level of evidence B-NR) (149). Percutaneous PVL closure often requires general anesthesia and the guidance of TEE, which undoubtedly brings more risks. Therefore, the application of ICE in PVL closure is of great significance. Ruparelia et al. conducted a retrospective study on the efficacy and safety of ICE-guided PVL closure (150). The results of this study showed that the success rate of ICE-guided PVL closure was 77.8%, which was similar to that of TEE, and there were no ICE-related complications. In postoperative follow-up, 78.6% of patients had improvement in heart failure symptoms without persistent hemolysis. There was no death within 30 days after surgery, and the 1-year survival rate after surgery was 71.4%. The results of this study suggest that ICE-guided PVL closure is safe and effective.

Intracardiac echocardiography imaging can locate the PVL intraoperatively, guide transseptal puncture, assist in device selection, determine residual leakage, and identify perioperative complications. For aortic PVL, the proposed transfemoral approach should be used for closure. For mitral PVL, if the PVL is close to the lateral side (6–10 o’clock direction of the atrial view of the mitral valve), use the antegrade approach of transseptal puncture for closure, and if the PVL is close to the septal side (2–4 o’clock direction of the atrial view of the mitral valve), use the retrograde approach for closure. For most patients, the placement of the ICE catheter in the right atrium can meet the imaging requirements of the operation, and very few patients require placement of the ICE catheter in the right ventricle or even the left atrium. For patients with failed PVL closure under ICE guidance, a repeated closure or other treatments can be considered. Although disposable catheters increase surgical costs, ICE-guided PVL closure avoids the need for anesthesiologists and reduces the time of surgery. There are currently no reports on PVL closure after ICE-guided TAVR. As most ICEs on the market can only assess regurgitation bundles by 2D and color Doppler, and due to the safety concerns associated with catheter placement, their clinical application is still greatly limited. The recent advent of real-time 3D/volumetric imaging can contribute to the further display of anatomical structures by ICE, thus improving the success rate of the operation, with good application prospects.

Experts’ recommendation: (1) The application of ICE in aortic and mitral PVL is worth recommending, especially in patients intolerant to TEE examination; (2) 3D/4D ICE is more helpful in PVL intervention.

## Others

### Application of intracardiac echocardiography in left atrial appendage closure

In LAAC, ICE is increasingly favored by surgeons and patients for its more flexible and convenient operation, richer and more comprehensive viewing angles, improved tolerance and safety, less X-ray exposure and contrast agent dosage compared with TEE. Almost all patients can tolerate ICE-guided LAAC under local anesthesia. The specific process is as follows (Supplementary material). It focuses on the multi-angle scanning and evaluation of the LAA. Combining the flexible operation of ICE catheter with the systematic evaluation of LAA, the 3D electroanatomical mapping system can even achieve zero X-ray and zero-contrast agent LAAC (24).

As ICE avoids the adverse effects and esophageal injury due to TEE probe entering the esophagus, and is well tolerated by patients, LAAC can be completed under local anesthesia. After the ICE catheter reaches the right atrium, three conventional sites, namely the right atrial body, right ventricular outflow tract and proximal segment of the coronary sinus, are usually recommended for layer-by-layer scanning of the LA/LAA to rule out the possible presence of atrial and atrial appendage thrombi, of which the right ventricular outflow tract is the best scanning site to rule out thrombi. In LAAC, if ICE is used to guide the transseptal puncture, the method for judging the level of puncture point is as follows: Select the site where the membrane at the lower end of the interatrial septum is close to the muscle; the method for judging the anteroposterior position of puncture point: The ICE sector is in the same plane as the puncture needle tip and the left superior pulmonary vein (LSPV) ridge site. After successful puncture of the interatrial septum, under the guidance of X-ray, adjust the direction of the ICE catheter tip, and deliver the ICE catheter into the LA along the direction of the guidewire; or deliver along the location of the septal marker point in a 3D electroanatomical system.

**FIGURE 10:**
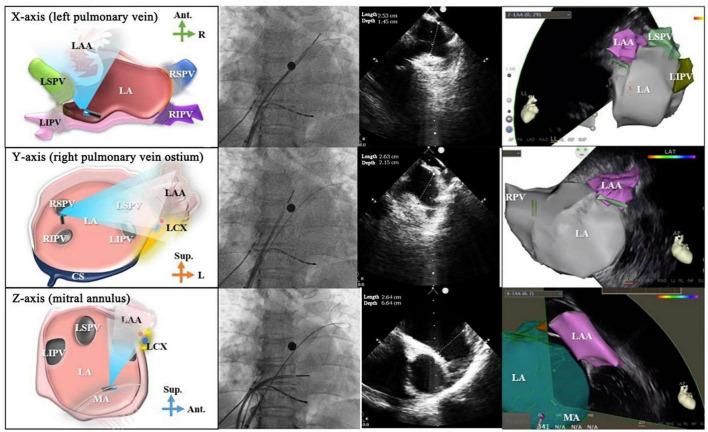
Multi-angle left atrial appendage measurement. From the top to bottom: schematic diagrams for atrial appendage assessment, X-ray images, ultrasound images and 3D electroanatomical diagrams from *X* axis, *Y* axis, and *Z* axis. The projection position of the X-ray is AP. The first column shows the schematic diagrams, the second column shows the effect of X-ray, the third column shows the images of ICE, and the fourth column shows the images of 3D mapping system. LAA, left atrial appendage; LSPV, left superior pulmonary vein; LIPV, left inferior pulmonary vein; LA, left atrium; RSPV, right superior pulmonary vein; RIPV, right inferior pulmonary vein; LCX, left circumflex artery; Ant., anterior; Sup., superior; R, right; L, left; MA, mitral annulus; RPV, right pulmonary vein.

The LAA is a 3D structure in the cardiac cavity, so its dimensions should be evaluated in three dimensions. The ICE catheter delivered into the LA allows close and multi-angle scanning of the LAA, avoiding the limitations of TEE application in patients with cardiac transposition and atrial appendage variations. Multi-angle assessment is the key point for ICE-guided LAAC. Routinely long-axis layer-by-layer scanning can be performed through three anatomical positions that are orthogonal, namely X axis (left pulmonary vein), Y axis (ostium of right pulmonary vein) and Z axis (mitral annulus) (as shown in Figure 10). The diameter of LAA opening and landing zone and effective working depth can be measured to better adapt to different anatomical structures and axial directions of different atrial appendages. The position, closure effect, compression and stability of the closure device can be evaluated in three dimensions after the closure device is expanded (151).

**FIGURE 11:**
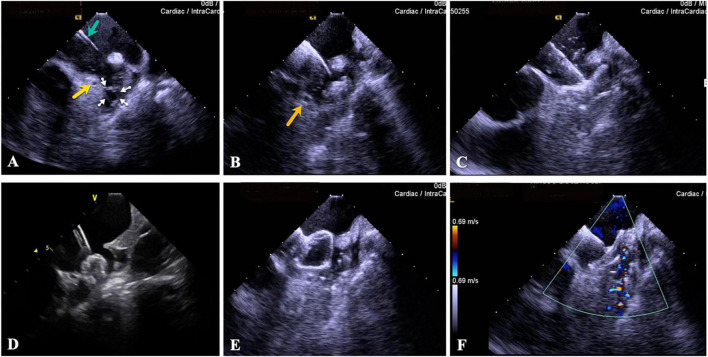
Process of ICE-guided LAAC. **(A)** Send the delivery sheath to the ostium of LAA (green arrow), and the pigtail catheter to the inside of LAA (small white arrow), and note the position of the LCX (yellow arrow). **(B)** Expand the closure device fixation disk, and note that it should be medial to the LCX (orange arrow). **(C)** Expand the closure disk. **(D)** Slightly withdraw the closure device (disk). **(E)** Pull test. **(F)** There is no residual shunt at the edge of the closure device as assessed by color Doppler flow monitoring at the end of closure. ICE, intracardiac echocardiography; LAA, left atrial appendage; LCX, left circumflex artery.

Place the ICE catheter in the LSPV and with a P curve, with the sector pointing to the LAA. Send the pigtail catheter into LAA, and an obvious catheter marker can be seen in the ultrasonic view, based on which the landing zone and left circumflex artery (LCX) are located. Send the delivery sheath into the ostium of LAA under the guidance of the pigtail catheter (as shown in Figure 11A). After the tip of the sheath entering the LAA reaches the required depth for closure, withdraw the pigtail catheter, and send the closure device (or fixation disk) to the LAA landing zone. Expand the closure device (Watchman closure device) or fixation disk (ACP or LAmbre closure device), and note that the site bearing the maximum force should be medial to the LCX (as shown in Figure 11B). Continue to expand the closure disk (ACP or LAmbre closure device, as shown in Figure 11C). If necessary, slightly or partially withdraw the closure device to adjust the position of the closure device (as shown in Figure 11D). After the closure device is at the desired position, perform a pull test under the guidance of ICE (as shown in Figure 11E). Finally perform color Doppler to examine whether there is residual shunt at the edge of the closure device (as shown in Figure 11F).

After the closure device (disk) is expanded, ICE is applied to evaluate the closure effect (including the position of closure device, closure tightness and stability) layer by layer from multiple angles. After the release criteria of each closure device (e.g., Watchman closure device should meet PASS principle) are met, completely release the closure device, and then perform ICE examination again from multiple angles to evaluate the release effect of the closure device, avoid the displacement of the closure device and the effect of closure device on adjacent structures (such as pulmonary vein and mitral valve), and observe whether there is pericardial effusion. If the release criteria are not met, withdraw (fully withdraw/partially withdraw/slightly withdraw) the closure device, adjust the position and expand again, and re-evaluate the expanding effect.

It should be noted that: (1) For AF patients who meet the indications for LAAC, after successful puncture of the interatrial septum under the guidance of ICE, it is recommended that the ICE catheter be delivered into the LA or LSPV through the transseptal puncture point and the layer-by-layer scanning through “three axes and six directions” that are orthogonal from three anatomical marks be performed to assess the shape, size, and anatomical structure of the LAA. (2) A closure device of appropriate size should be selected according to the width and available depth of the LAA opening measured by angiography and ICE. After implantation of the closure device, angiography and ICE can be carried out to confirm that there is no large amount of residual shunt around the closure device, and the final pull test can be performed to examine the anchorage of the closure device.

### Application of intracardiac echocardiography in pregnant patients

Management of arrhythmias during pregnancy is an important challenge, and arrhythmias during pregnancy will lead to many adverse effects on the mother and fetus. There are many drawbacks associated with antiarrhythmic drugs, e.g., limited drug options, significant adverse reactions, and ineffective treatment. Despite a small radiation exposure, the conventional catheter ablation still brings risks to pregnant women and fetuses, which limits its use in special populations (152).

In recent years, as 3D electroanatomical mapping systems and ICE technique developed, completely zero X-ray catheter ablation has become feasible and in many institutions routine, which makes catheter ablation possible for the treatment of arrhythmias during pregnancy, including conventional supraventricular tachycardia and ventricular premature beats, as well as complex arrhythmias such as atrial fibrillation and ventricular tachycardia. ICE technique combined with 3D electroanatomical mapping system allows visualization of the multi-electrode catheter, facilitates electrode placement, and makes subsequent ablation of the left heart system viable after the ICE-guided transseptal puncture is completed (153–155). In addition to catheter ablation during pregnancy, ICE can also be used during pacemaker implantation and zero X-ray therapy for some congenital heart diseases. However, the indications for zero X-ray interventional operations during pregnancy should be strictly specified, and the success rates of these operations, complications, radiation exposure and other risks require continued evaluation and experience. For the sake of safety, zero X-ray operation may not always be viable, so surgeons should collaborate with the Gynecology and Obstetrics team and the anesthesiology team in planning procedures in this patient population.

### Application of intracardiac echocardiography in left ventricular assist device implantation

Intracardiac echocardiography is a viable option to guide LVAD implantation, especially when other imaging modes are not appropriate. However, to avoid serious iatrogenic complications, clinicians must understand the limitations of experience, the limitations of imaging, and the risks associated with this technique. In some special cases, X-ray, TTE, and TEE are clinically not indicated for LVAD implantation, while ICE can guide the LVAD implantation in such cases. Moreover, compared with TTE and TEE, ICE can be used in patients requiring positive pressure ventilation and intubation. However, for ICE in the chambers of the heart, soft guidewire wrapping is possible during transaortic implantation of LVAD, in which case excessive tension of the guidewire may lead to a potential complication (vascular perforation). Moreover, ICE is sometimes challenging to image the descending aorta due to its limited spatial resolution, which may bring additional risks (156).

Intracardiac echocardiography can be used as an alternative or supplement to TEE for perioperative and postoperative management of LVADs. There are case reports where the device structure, hemodynamics and related complications after ICE-guided LVAD implantation are evaluated (157–159). Compared with TTE, TEE, or 3D computed tomography, ICE features high success rate of treatment, no need for general anesthesia, no need for radiation exposure, and shortened recovery time (5, 159) (Flow chart 10 shown in Supplementary materials).

### Application of intracardiac echocardiography in myocardial biopsy

Intracardiac echocardiography-guided endomyocardial biopsy (EMB) is an interesting application of ICE. ICE enables precise localization for biopsy, reduces the risks associated with operations, and reduces the need for diagnostic thoracotomy, and is more maneuverable in selected cases (160). With this emerging technique, the diagnostic yield of biopsy is significantly improved, with fewer complications (161).

A series of patients with right ventricular mass undergoing ICE-guided EMB demonstrated safety and efficacy (60). The diagnosis of both cardiac metastatic tumors and primary cardiac tumors is based on histopathology. According to this series of cases, EMB is a valuable tool for preoperative diagnosis and surgical planning of intracardiac masses suspected of tumors, while ICE is worthy of further attention for its ability to accurately locate cardiac structures and guide biopsy sampling in the target area. ICE can be used to guide EMB of cardiac masses. Allowing a correct positioning of the bioptome, ICE reduces the procedure-related risks and the need for a diagnostic open-chest procedure, reserving the more invasive approach to selected cases (160).

Electroanatomical mapping combined with ICE-guided EMB is feasible in patients with suspected arrhythmic cardiomyopathy and ventricular arrhythmia of unknown origin. This method can significantly improve the positive rate of biopsy and reduce complications (161) (Flow chart 11 shown in Supplementary materials).

### Application of intracardiac echocardiography in hypertrophic obstructive cardiomyopathy

Radiofrequency ablation of the interventricular septum is currently a novel surgical technique for hypertrophic obstructive cardiomyopathy, and its feasibility has been demonstrated in some studies (162, 163). Accurate localization of the anterior mitral leaflet and the ventricular septal flapping area is the key to radiofrequency ablation of the interventricular septum. Previously, the localization of the target area during the interventricular septal ablation for hypertrophic cardiomyopathy was mainly achieved by TEE combined with CARTO system (164). Intraoperative continuous TEE monitoring is limited due to limited patient tolerance, and general anesthesia and esophageal intubation also bring additional risks such as gastroesophageal injury and aspiration (165). In addition, ICE is non-inferior to TTE in imaging the overall cardiac structure (166). Previous studies have demonstrated that ICE can provide detailed information on the anatomical structure of the interventricular septum (166). As an organic combination of ICE image and 3D positioning system image, CARTO-Sound clearly displays the mitral valve and interventricular septal flapping area, and can be used to guide the selective precise ablation of the tip. 3D models of left ventricle, left ventricle outflow tract, anterior mitral leaflet and aortic root are constructed by ICE, which can accurately trace the site of interventricular septum obstruction (anterior leaflet and interventricular septal flapping area), and intraoperatively monitor the contact of the ablation catheter and the ablation injury in real-time. Patients can benefit from ICE-guided interventricular septal ablation. A small-scale study showed that 20 patients with significantly symptomatic hypertrophic obstructive cardiomyopathy had aortic transvalvular pressure gradient of greater than 50 mmHg by preoperative resting TTE and were followed up for 6 months after interventricular septal ablation. In patients with interventricular septal hypertrophy and obstruction, short anterior mitral leaflet and normally positioned papillary muscles, the NYHA cardiac function classification was significantly improved, and left ventricle outflow tract pressure and pressure gradient were significantly reduced (100). In addition, the feasibility of ICE-guided interventricular septal ablation in hypertrophic cardiomyopathy has also been confirmed in some small-scale studies (162, 167), and it is worthy of further study.

### Application of intracardiac echocardiography in pulmonary arterial hypertension

In patients with aortic valve stenosis and pulmonary arterial pressure undergoing TAVR, the use of ICE instead of right heart catheterization for pulmonary arterial pressure monitoring is safe and feasible (168). The pulmonary arterial hypertension is mainly treated with drugs, while balloon atrial septostomy (BAS) can be performed as a bridging therapy or palliative therapy for poorly controlled patients awaiting lung transplantation (169, 170). Under the guidance of ICE, the surgeon can accurately locate the fossa ovalis and the surrounding anatomical structures, reducing the need for radiation. The feasibility of ICE-guided balloon or implantable device atrial septostomy has been demonstrated in studies (171–174). ICE-guided BAS combined with radiofrequency ablation has been shown in animal experiments to be effective in reducing spontaneous closure after atrial septostomy (175). ICE-guided AS is promising. Potts shunt has some advantages over AS in patients with severe pulmonary arterial hypertension who fail to respond to drug treatment: (1) It has a more reliable patency; (2) It does not lead to persistent hypoxemia in the upper part of the body, so the coronary artery and cerebral circulation are not affected. However, this procedure comes with high risks during the establishment of descending aorta and main pulmonary artery channels. In the future, 4D ICE real-time volumetric imaging and multiplanar reconstruction techniques can be used to guide the puncture, which may improve the operation safety, so that this operation can be further popularized and applied for the benefit of more patients. In addition, ICE can clearly show the right ventricular outflow tract, pulmonary valve, and proximal pulmonary artery. Therefore, ICE is also promising in percutaneous pulmonary artery sympathetic denervation ablation for pulmonary arterial hypertension. However, the use of ICE in pulmonary arterial hypertension needs to be further explored and validated in relevant clinical studies.

### Application of intracardiac echocardiography in lead extraction

With the increasing use of cardiac electronic implantable devices such as pacemakers, device-related complications, such as infection, thrombosis and device/lead failure, occur frequently. Transvenous lead extraction (TLE) is an important technique for managing cardiac electronic implantable device-related infections (176). In TLE, conventional fluoroscopy, TEE and preoperative CT imaging have their own limitations: The wear and residue of non-visualized components such as lead insulation layer cannot be identified under fluoroscopy, and the adhesion of leads to tissues cannot be assessed; CT imaging is incapable of real-time guidance and assessment; TEE is performed close to the back of the heart, so the images for other regions are of poor quality and reproducibility.

As a unique imaging technique, ICE can guide TLE or be combined with new techniques intraoperatively, and it is safe and feasible (177–180). ICE can evaluate the lead condition, thrombus, neoplasm, lead “ghost” and adhesion of leads to tissues under direct vision, and can also timely detect the hyperechogenicity around the lead (181) (LAEs, which are usually classified as thrombus or neoplasm, prevalent in three-quarters of patients undergoing lead implantation, and are found in the right atrium, tricuspid valve, right ventricle and superior vena cava) to prevent such complications as embolism; it is currently being explored to remove LAEs under the guidance of ICE. Preoperative evaluation of the adhesion of leads to vessels, valves, right atrial free walls, and right atrial appendages by ICE is of great significance, contributing to the identification of high-risk valves, risk assessment of lead extraction, tool selection, and early judgment of the need for surgical extraction or delayed extraction; ICE enables dynamic risk assessment intraoperatively, real-time monitoring of complications, accurate identification of false cardiac tamponade due to traction under TEE during lead extraction, and dynamic assessment of tricuspid valve function. It is of great significance to reduce postoperative complications and improve operation safety (Flow chart 12 shown in Supplementary materials).

Intracardiac echocardiography has been widely used in cardiac interventions such as radiofrequency ablation, and it is also suitable for TLE based on the said advantages. With the advancement of technique and the application of 3D ultrasound imaging, the imaging quality of ICE will be further improved in the future. Therefore, the application prospect of ICE-guided TLE is promising.

## Conclusion

Nowadays, ICE has been applied in a variety of cardiovascular interventional operations. In addition to the applications introduced above, ICE can also be used to guide the implantation of leadless pacemaker (182), left bundle branch pacing (183), etc. At the same time, for patients who have implanted pacemaker, zero-fluoroscopy ablation can also be completed with the assistance of ICE (184). However, more clinical evidence is needed to prove the feasibility of these operations.

Currently, catheter with a diameter of 8F has emerged, which will expand the application of ICE, such as cardiovascular interventional procedures in children and so on. With the application of 4D ICE, the improvement of image quality and the decrease of cost, ICE will be more widely used in the future.

## Limitations

Because there are few authoritative clinical studies related to ICE in the world, some of viewpoints in our consensus are lack of considerable clinical studies to support them, and they were based on the clinical experience of the experts or case reports, we don‘t use “I-III” and “a-c” to express evidence level but use “Recommend,” “Can be useful,” “Maybe useful,” “Not recommended,” (Table 2) which is also one of the reasons why our manuscript is consensus rather than a guideline.

As there are few expert consensuses related to ICE in the world, the flowcharts (in Supplementary material) in our manuscript are developed based on the experience of Chinese experts. It may not be applicable to medical centers in different countries and regions, but it can be used as a reference.

Some emerging technologies (such as 4D ICE and 8F catheter) related to ICE are only mentioned in this manuscript, and their clinical applications are not elaborated too much, because they are rarely used in China. We hope that we will have the opportunity to improve the expert consensus with the application of these technologies in the future.

## Author contributions

All authors listed have made a substantial, direct, and intellectual contribution to the work, and approved it for publication.
