# Supplementary figures and images for: Intracardiac echocardiography Chinese expert consensus
Source: Front Cardiovasc Med. 2022 Oct 6;9:1012731. doi: 10.3389/fcvm.2022.1012731 (PMC9584059; doi:10.3389/fcvm.2022.1012731)

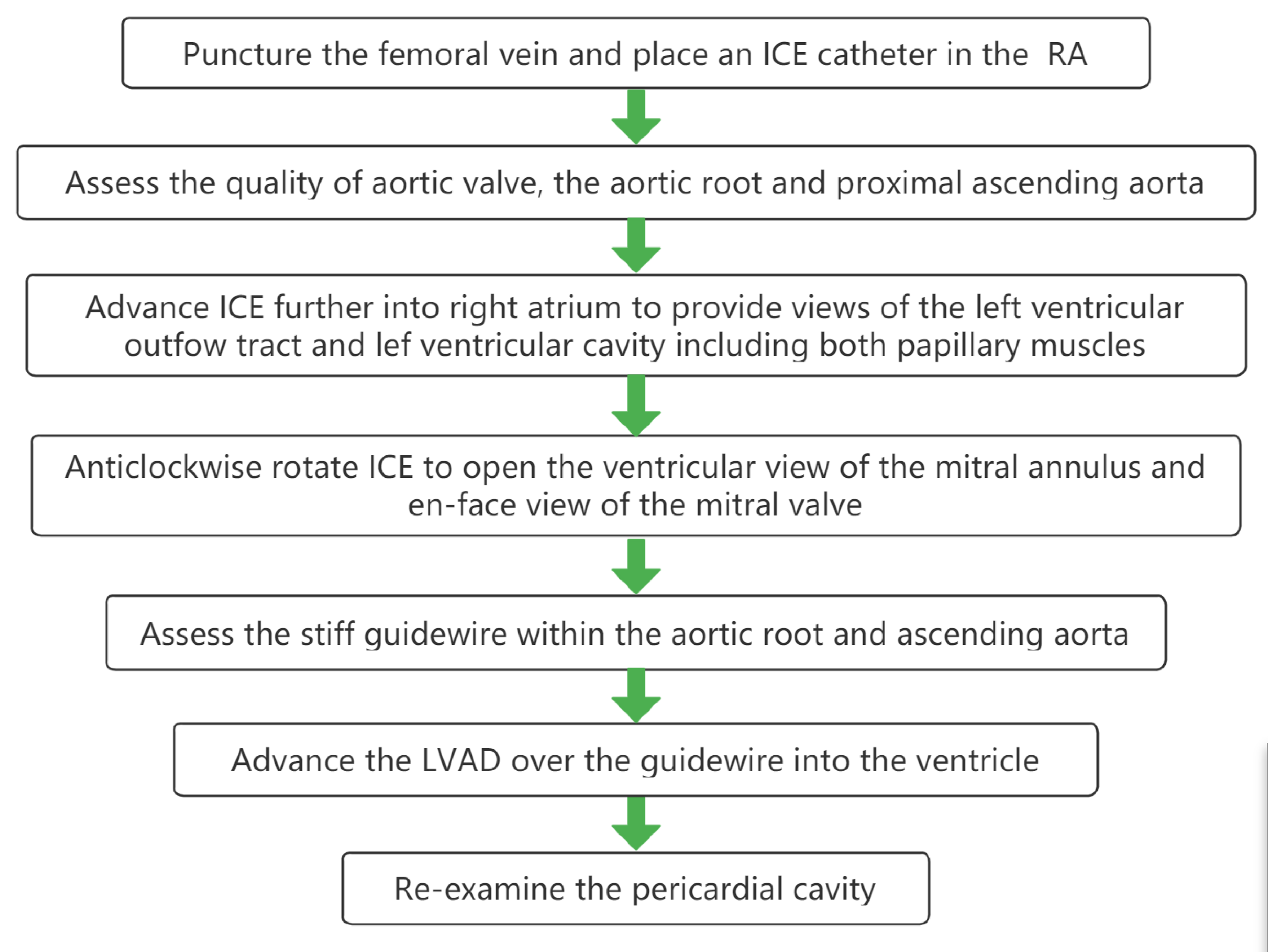

Supplement: Supplementary file 1 [file Presentation_1.zip › Presentation 1/flowchart 10ú║ LVAD.tif]

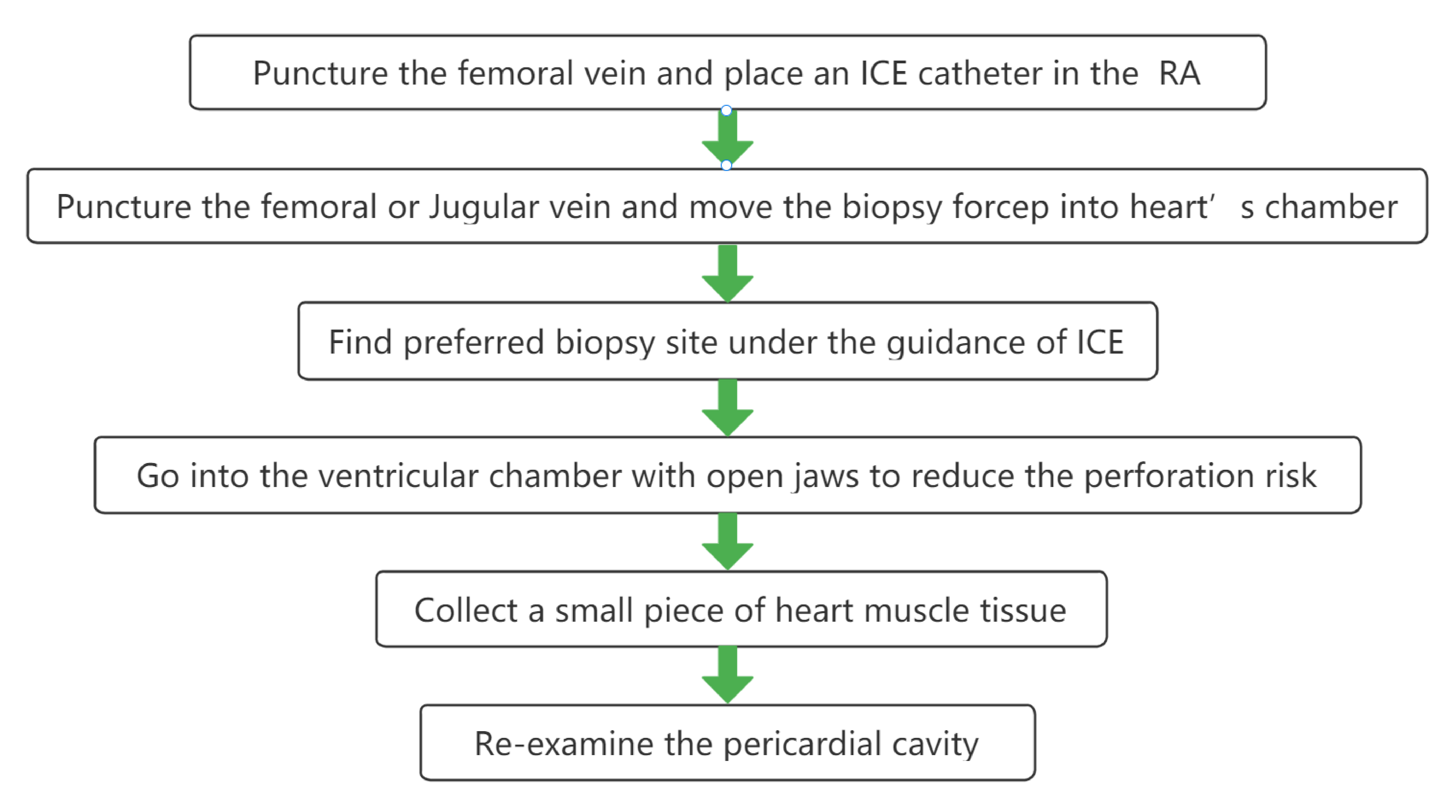

Supplement: Supplementary file 1 [file Presentation_1.zip › Presentation 1/flowchart 11ú║ Myocardial biopsy.tif]

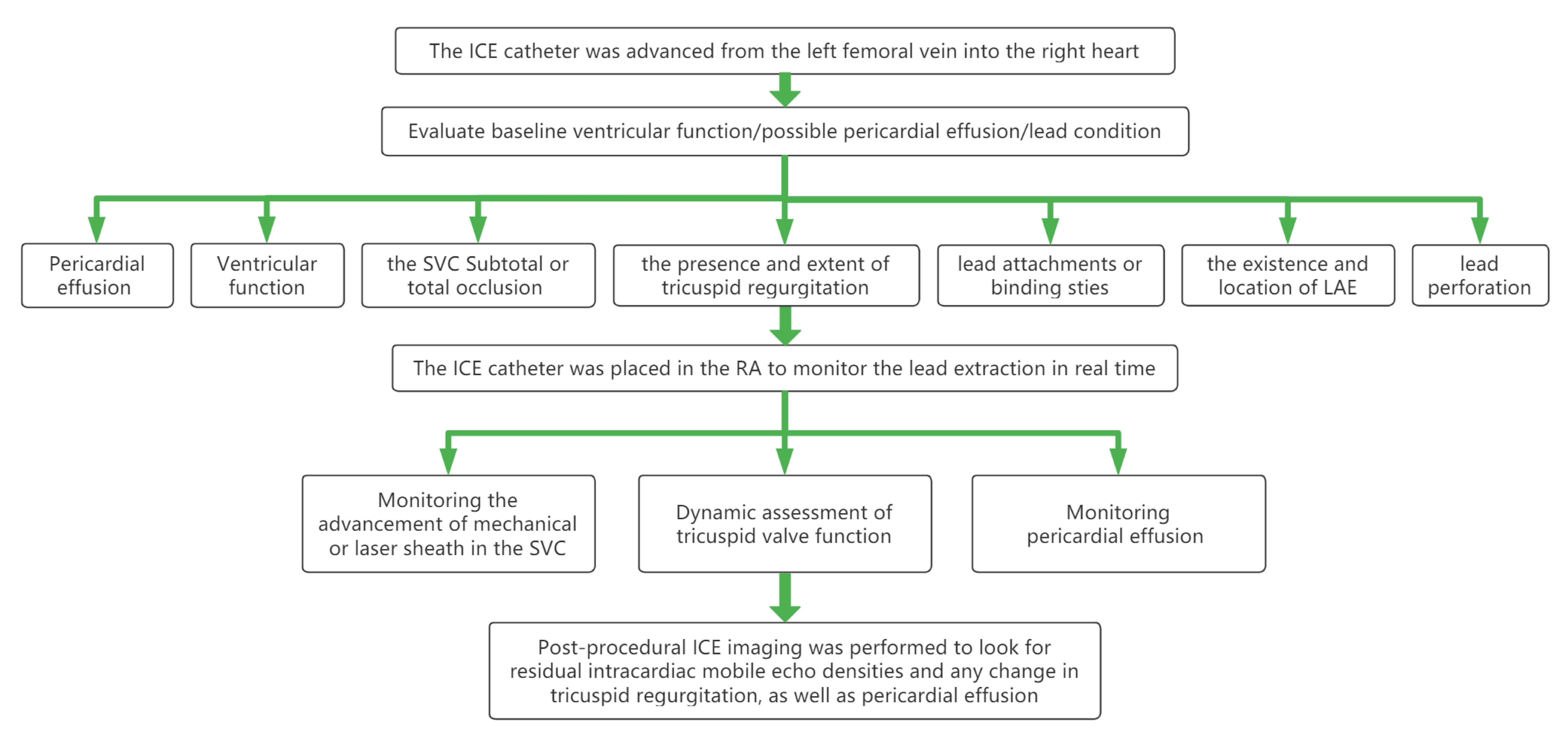

Supplement: Supplementary file 1 [file Presentation_1.zip › Presentation 1/flowchart 12ú║ lead extraction.tif]

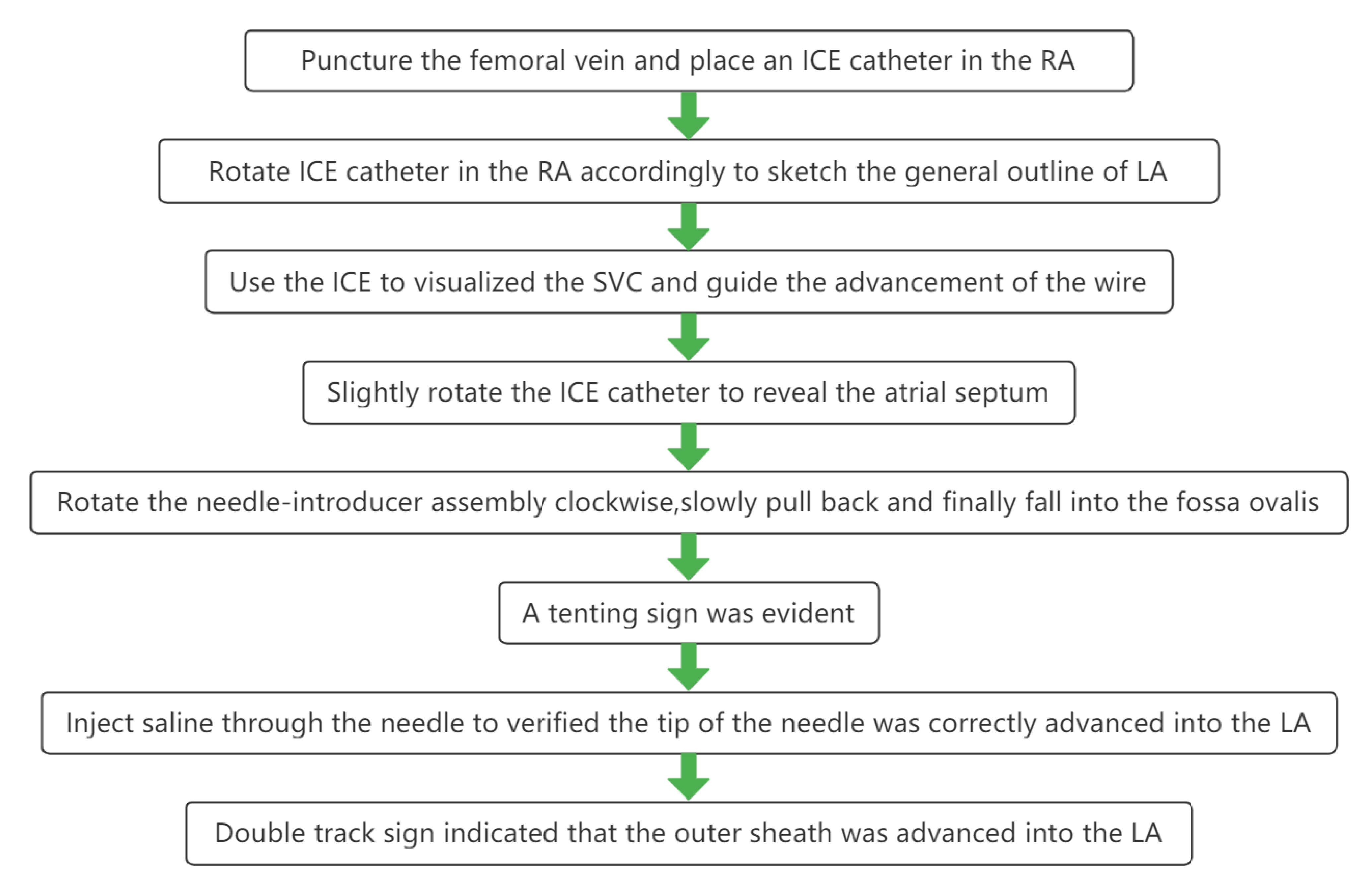

Supplement: Supplementary file 1 [file Presentation_1.zip › Presentation 1/flowchart 1ú║ Transseptal Puncture.tif]

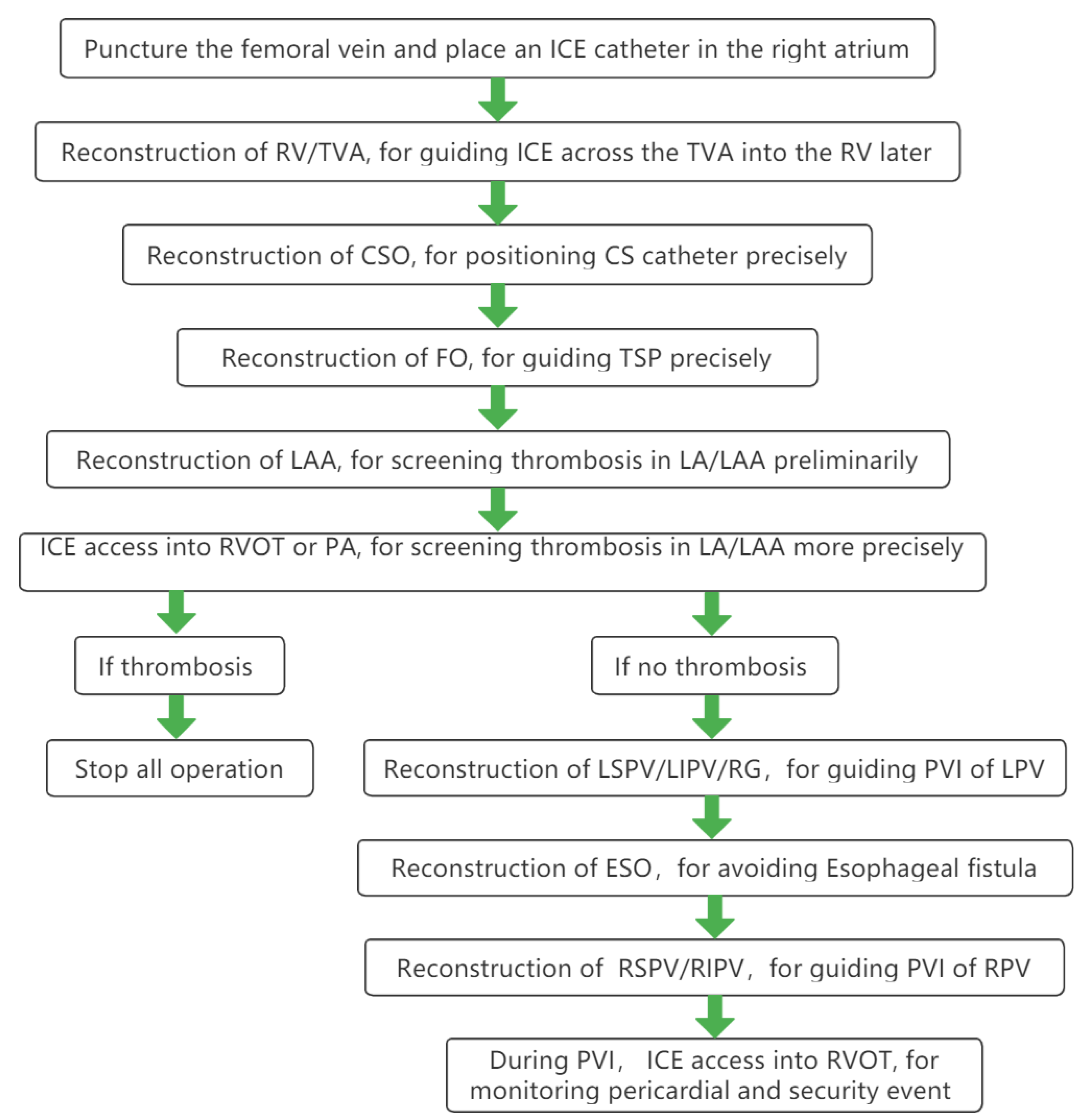

Supplement: Supplementary file 1 [file Presentation_1.zip › Presentation 1/flowchart 2ú║ AF ablation.tif]

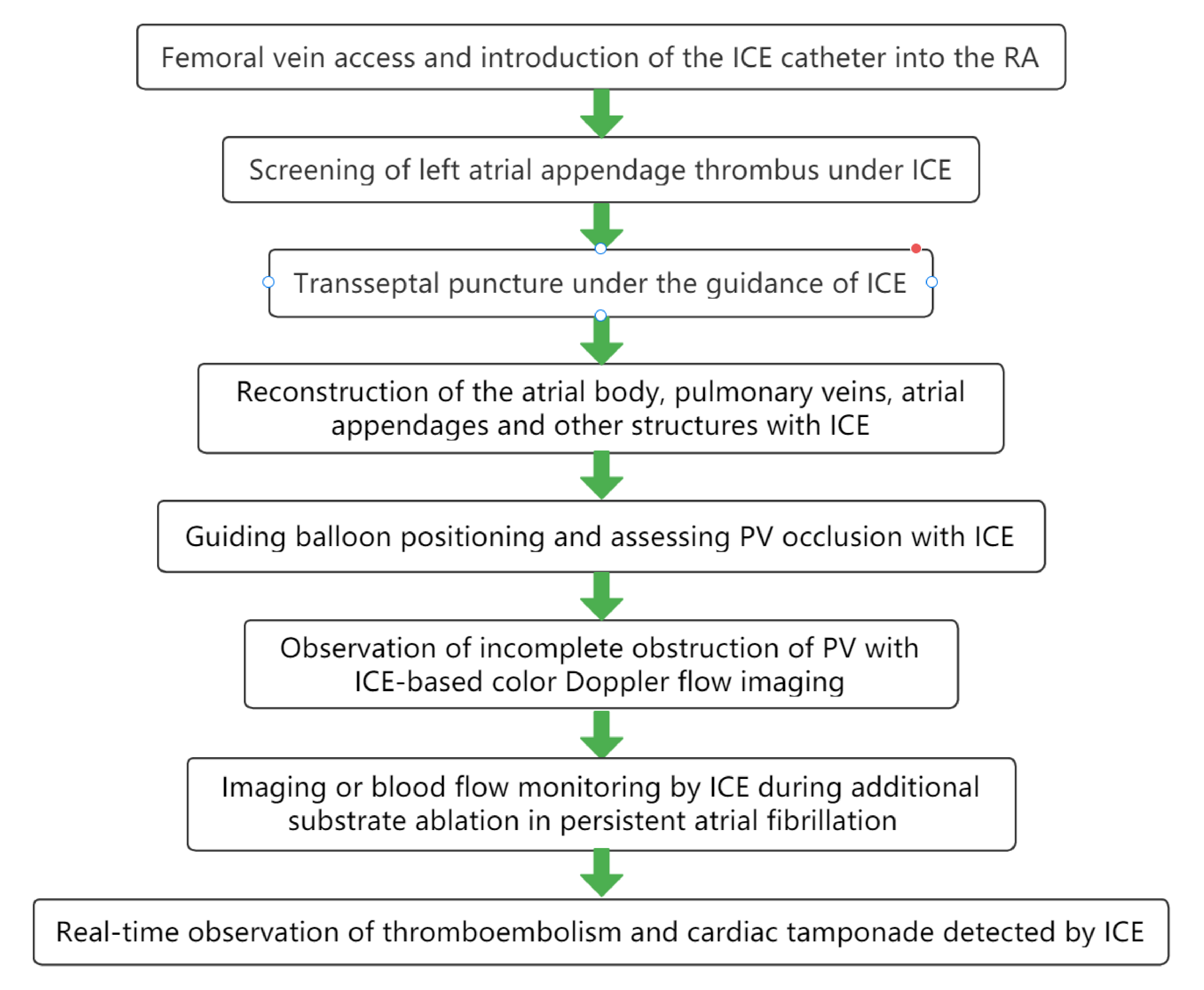

Supplement: Supplementary file 1 [file Presentation_1.zip › Presentation 1/flowchart 3ú║ Cryoballoon ablation.tif]

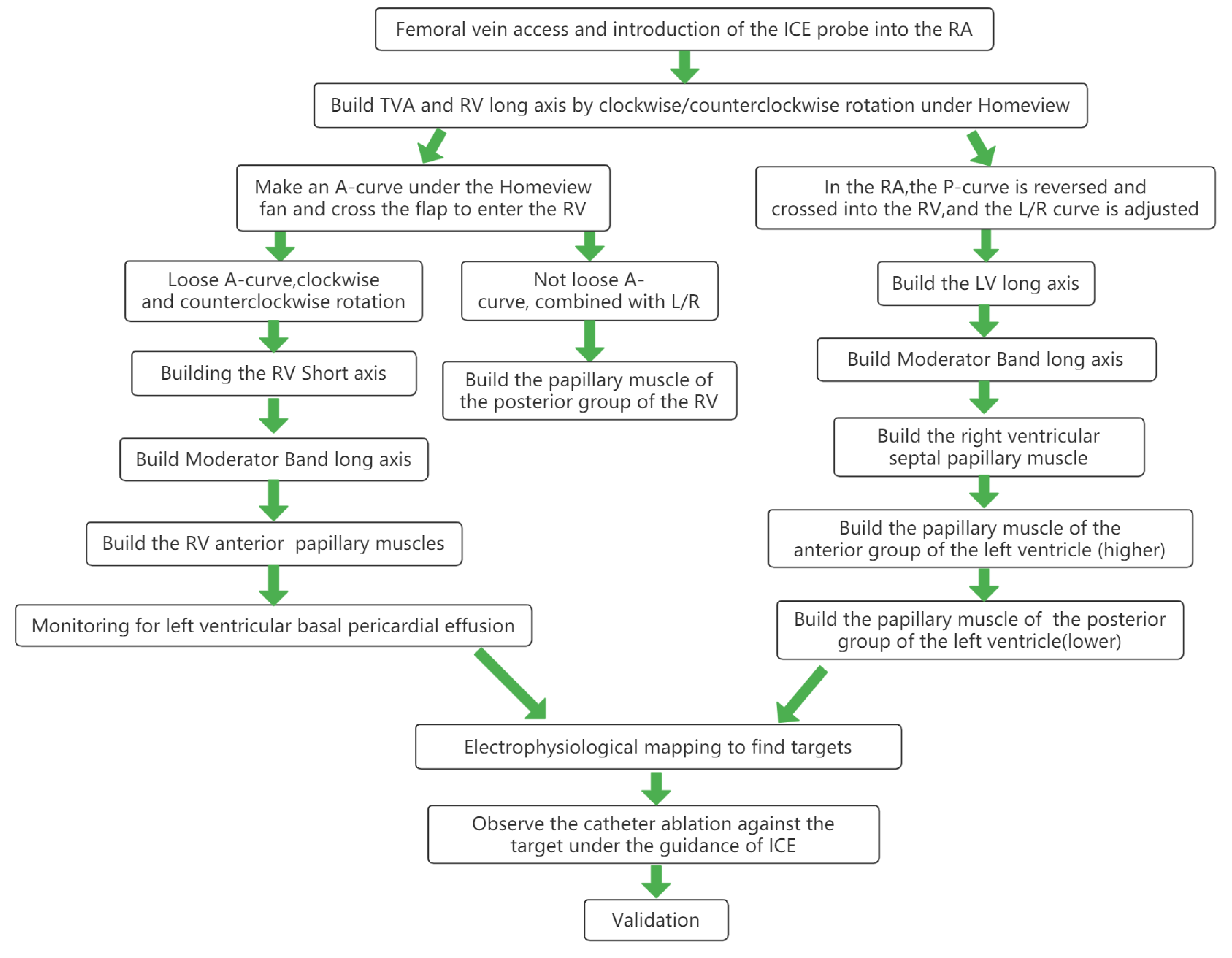

Supplement: Supplementary file 1 [file Presentation_1.zip › Presentation 1/flowchart 4ú║Ventricular Arrhythmia ú¿papillary muscleú⌐.tif]

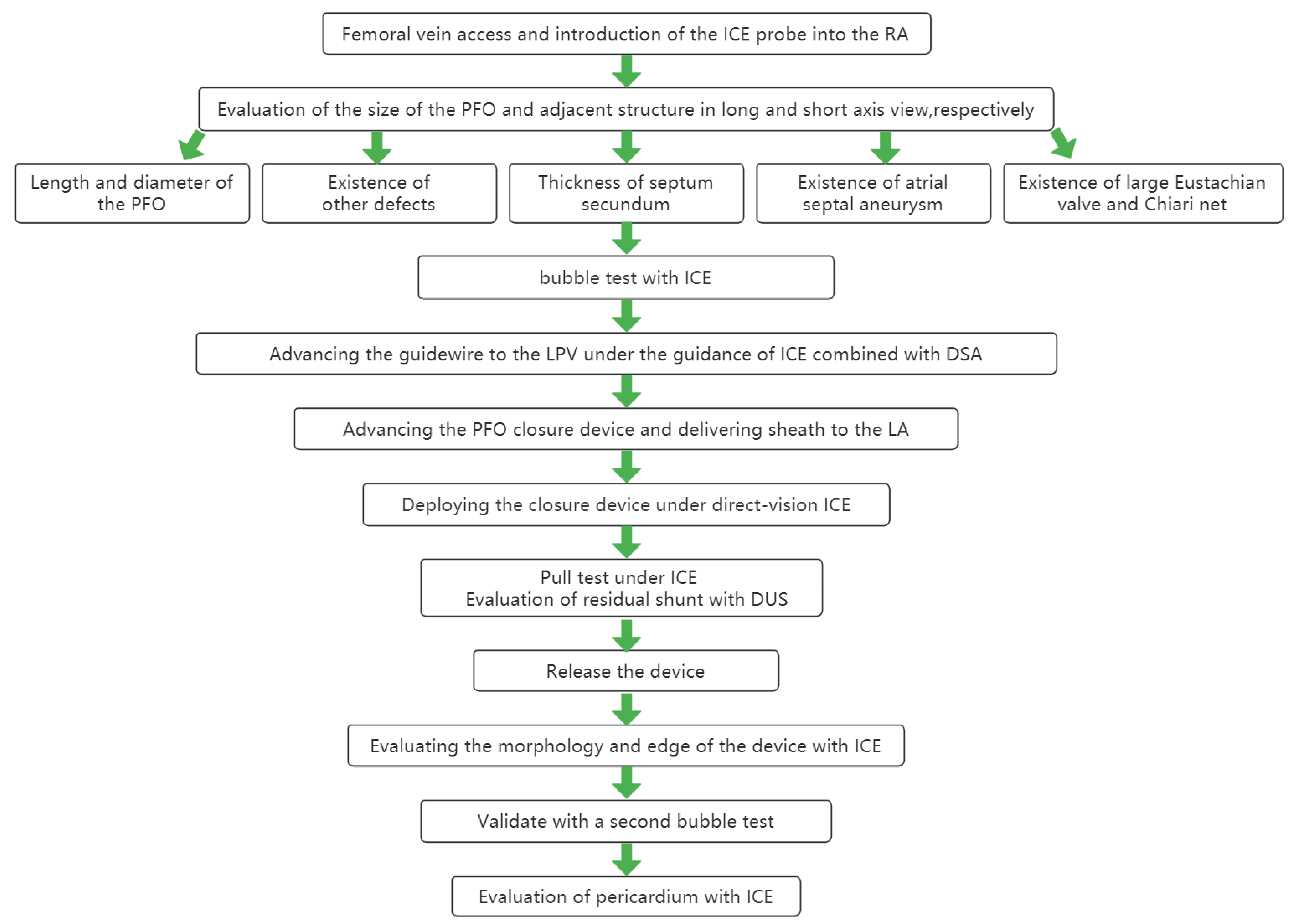

Supplement: Supplementary file 1 [file Presentation_1.zip › Presentation 1/flowchart 5ú║PFO closure.tif]

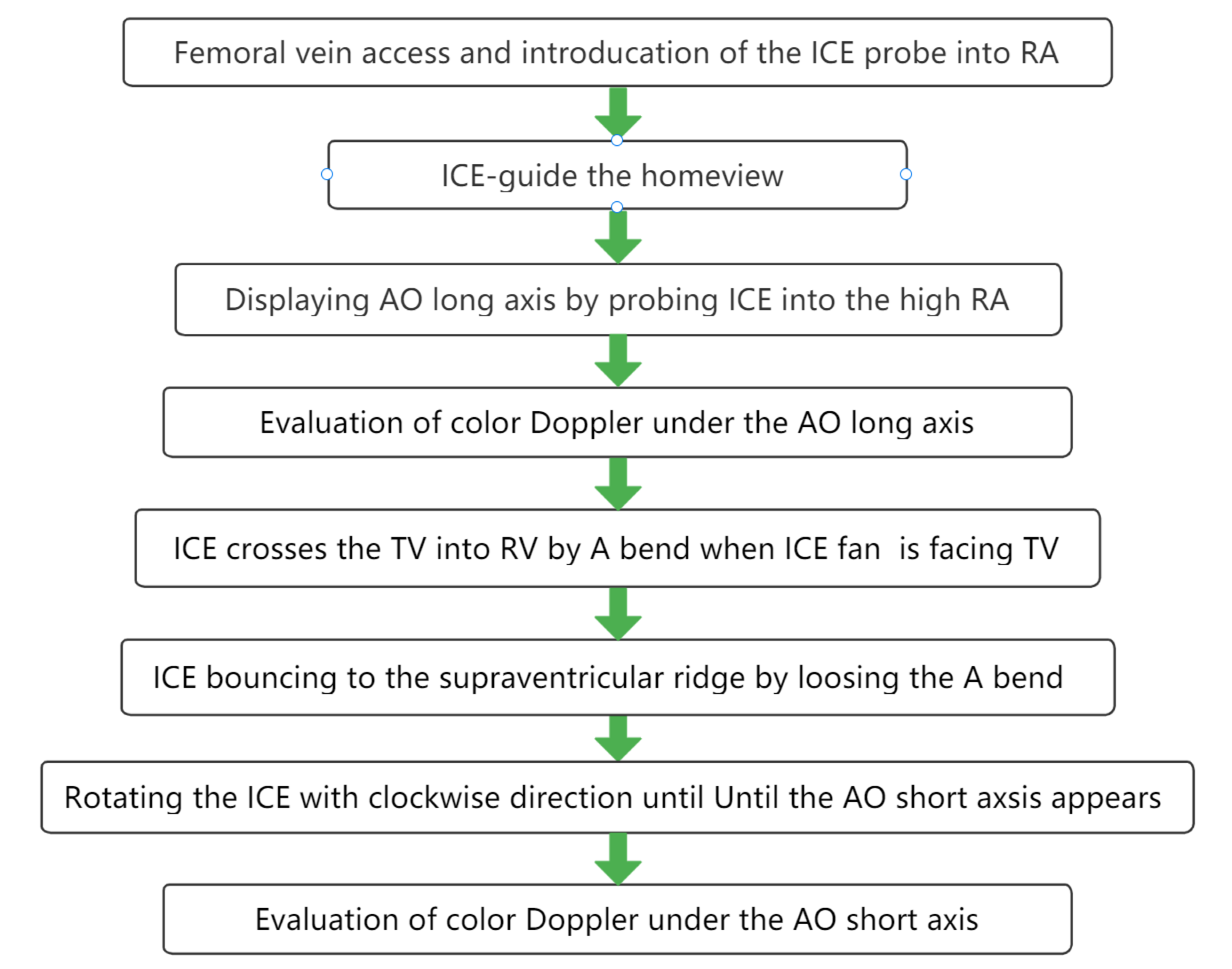

Supplement: Supplementary file 1 [file Presentation_1.zip › Presentation 1/flowchart 6ú║ TAVR.tif]

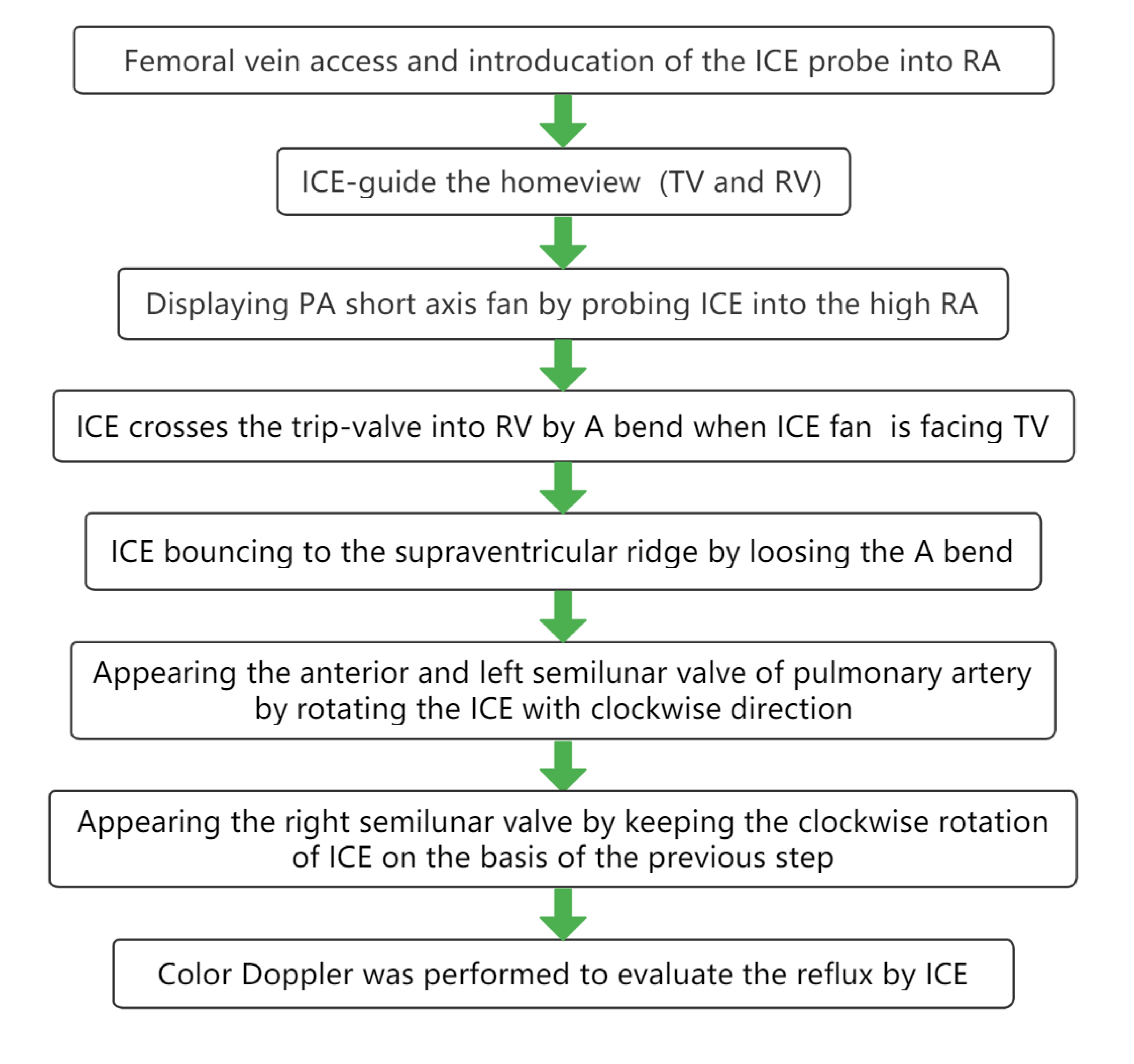

Supplement: Supplementary file 1 [file Presentation_1.zip › Presentation 1/flowchart 7ú║Pulmonary Valve Intervention.tif]

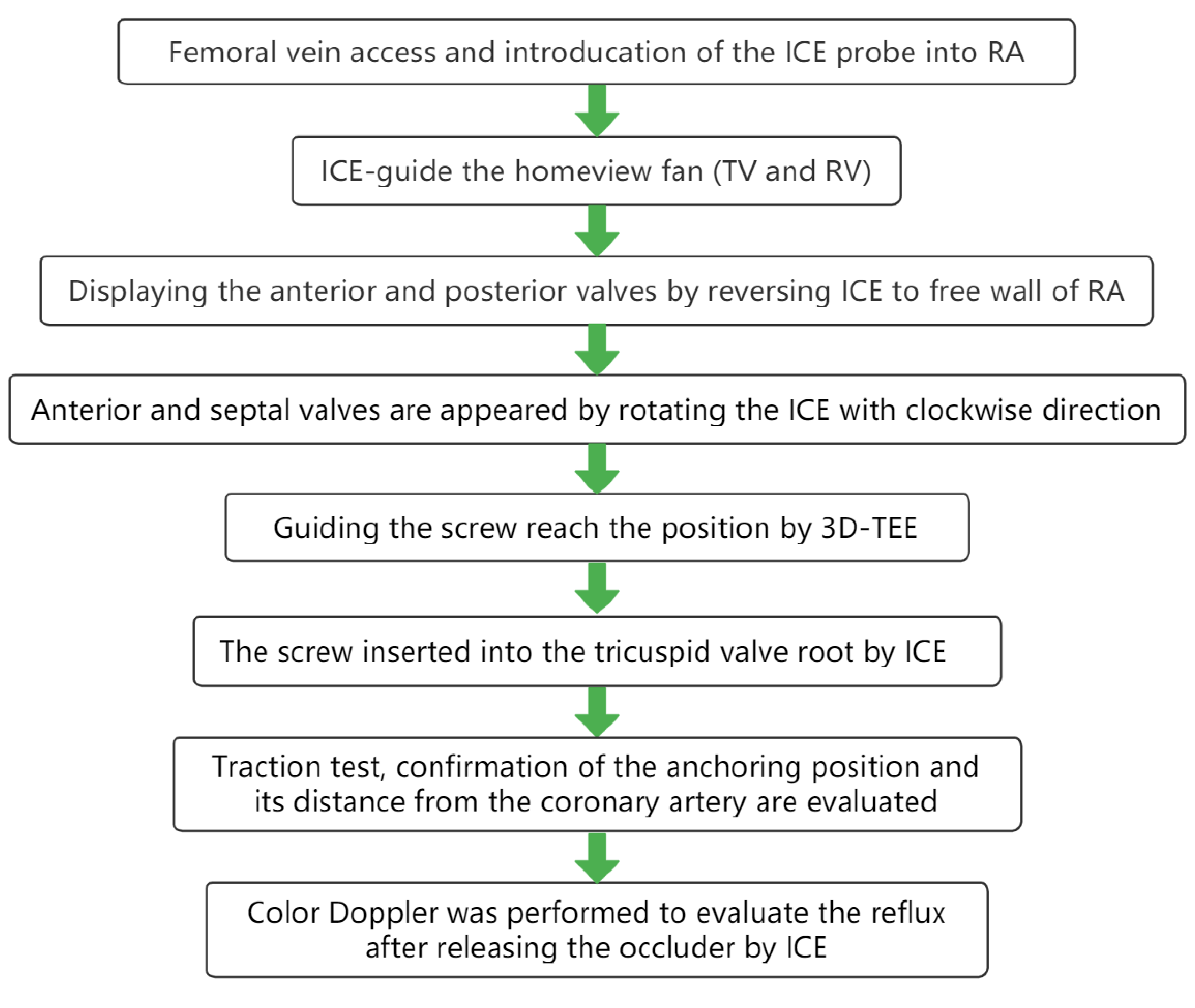

Supplement: Supplementary file 1 [file Presentation_1.zip › Presentation 1/flowchart 8ú║TR intervention by k-clip.tif]

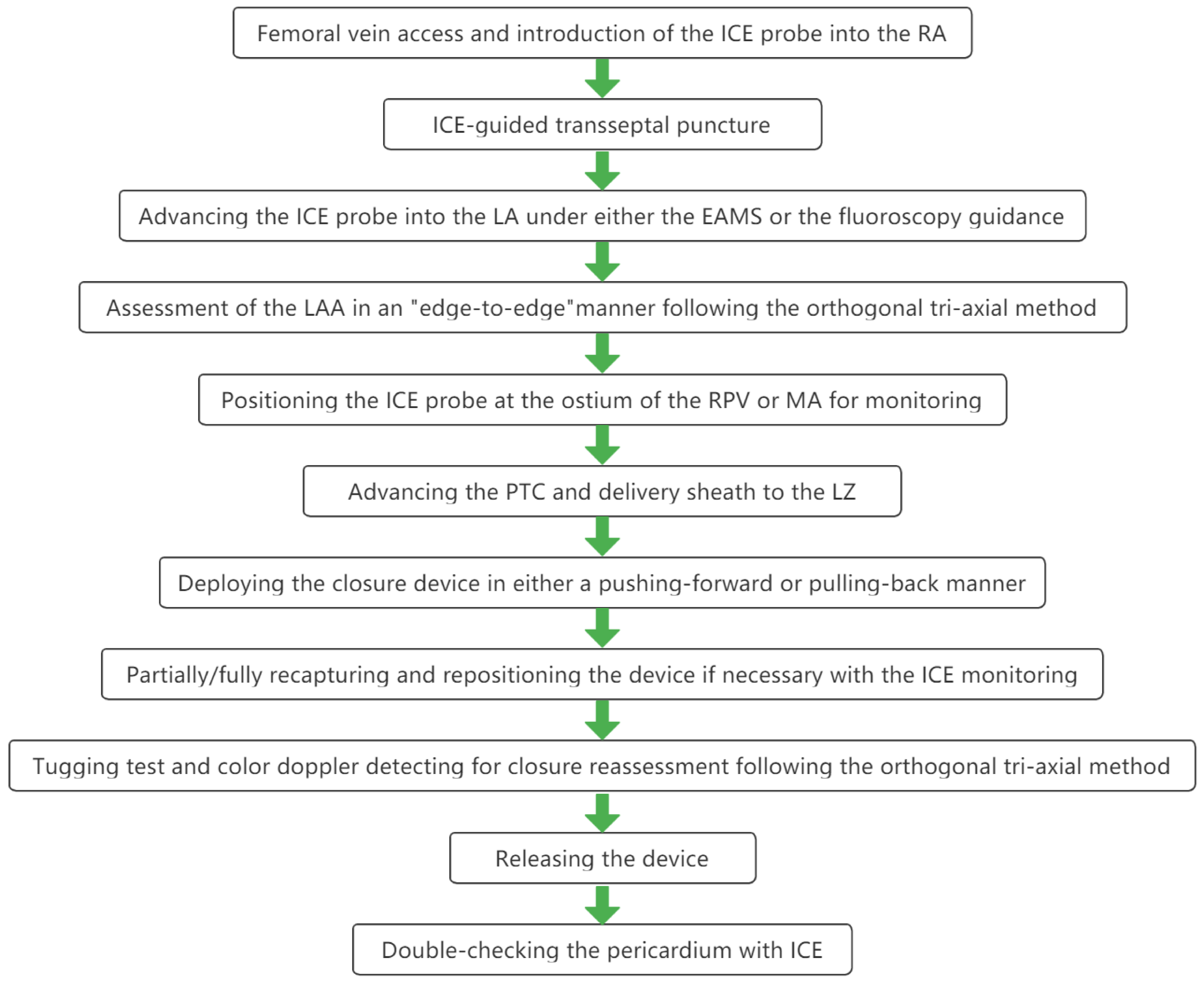

Supplement: Supplementary file 1 [file Presentation_1.zip › Presentation 1/flowchart 9ú║ LAAC.tif]
